# Deciphering the genetic basis of root morphology, nutrient uptake, yield, and yield-related traits in rice under dry direct-seeded cultivation systems

**DOI:** 10.1038/s41598-019-45770-3

**Published:** 2019-06-27

**Authors:** Nitika Sandhu, Sushil Raj Subedi, Vikas Kumar Singh, Pallavi Sinha, Santosh Kumar, S. P. Singh, Surya Kant Ghimire, Madhav Pandey, Ram Baran Yadaw, Rajeev K. Varshney, Arvind Kumar

**Affiliations:** 1 Rice Breeding Platform, International Rice Research Institute, Metro Manila, Philippines; 2 International Rice Research Institute, South Asia Hub, ICRISAT, Patancheru, Hyderabad India; 3 Center of Excellence in Genomics and System Biology, International Crops Research Institute for the Semi-Arid Tropics (ICRISAT), Patancheru Hyderabad, India; 4 ICAR Research Complex for Eastern Region, Patna, Bihar India; 5 Bihar Agricultural University, Sabour, Bhagalpur, Bihar India; 6 Agriculture and Forestry University, Rampur, Chitwan Nepal; 7 National Rice Research Program, Hardinath, Nepal; 8 Punjab Agricultural University, Ludhiana, India

**Keywords:** Genomics, Genome-wide association studies

## Abstract

In the face of global water scarcity, a successful transition of rice cultivation from puddled to dry direct-seeded rice (DDSR) is a future need. A genome-wide association study was performed on a complex mapping population for 39 traits: 9 seedling-establishment traits, 14 root and nutrient-uptake traits, 5 plant morphological traits, 4 lodging resistance traits, and 7 yield and yield-contributing traits. A total of 10 significant marker-trait associations (MTAs) were found along with 25 QTLs associated with 25 traits. The percent phenotypic variance explained by SNPs ranged from 8% to 84%. Grain yield was found to be significantly and positively correlated with seedling-establishment traits, root morphological traits, nutrient uptake-related traits, and grain yield-contributing traits. The genomic colocation of different root morphological traits, nutrient uptake-related traits, and grain-yield-contributing traits further supports the role of root morphological traits in improving nutrient uptake and grain yield under DDSR. The QTLs/candidate genes underlying the significant MTAs were identified. The identified promising progenies carrying these QTLs may serve as potential donors to be exploited in genomics-assisted breeding programs for improving grain yield and adaptability under DDSR.

## Introduction

Rice, one of the staple food crops, is a large consumer of freshwater. Irrigated rice accounts for approximately 55% of the global harvested area and contributes 75% of global rice production with 25% use of global agricultural freshwater^1^. Farmers are witnessing severe problems associated with the scarcity of water, labor, and resources with changing climatic conditions. Dry direct-seeded rice (DDSR) can effectively address the problem of water-labor shortage in both rainfed and irrigated areas through reduced use of water for land preparation. In addition, the DDSR system saves water through better irrigation management and the introduction of mechanized practices for sowing, weed control, harvesting, and threshing.

The key challenges under direct-seeded rice cultivation systems include poor crop stand, low yield^2^, weeds^3^, poor adaptability, reduced nutrient uptake (especially of phosphorus, nitrogen, and iron)^4^, and lodging^5^. The unavailability of DDSR varieties suitable to yield more under the increasing water and labor shortage scenario demand the development of DDSR varieties with improved yield, weed competitivness, and better root traits that enhance nutrient uptake^2^. The inefficient uptake of water and nutrients under non-flooded conditions resulting from the poor root structure^4,6,7^ of puddled transplanted rice needs to be addressed through traits and varietal development programs. The limited adaptability of the roots to adjust quickly to the frequent changes in soil conditions from flooded to aerobic and vice-versa is also a major constraint to water-nutrient uptake under DDSR. The plant root system architecture is reported to be dependent on water-nutrient availability, uptake, and signaling^4,6–8^. A clear picture of an ideal root system required for higher water-nutrient uptake under DDSR may offer a real possibility of grain yield improvement under DDSR. Non-uniform and fast emergence together with significant seedling death just after seeding and very low weed competitiveness are important factors that determine reduced yield under DDSR. Plant morphological traits such as leaf area index; leaf size; leaf insertion angle, shape, and thickness^9^ contributing to photosynthesis^10^; flowering time^11–13^; growth rate; lodging resistance^4,14^; root traits that enhance nutrient uptake, especially nitrogen (N), phosphorus (P), and iron (Fe)^4^; tillering ability; panicle length; and spikelet fertility are important traits that determine crop productivity under DDSR.

To date, no detailed study has been conducted on the genetic basis associated with plant morphological traits and root system architecture associated with nutrient uptake, yield, and yield-contributing traits^15^ under DDSR. There is thus an urgent need to decipher an appropriate plant and root system architecture for improving nutrient uptake, grain yield, and adaptability under DDSR.

Emerging genomic technologies may allow the systematic investigation on a genome-wide scale to harness genetic diversity^16,17^ and associations leading to rice yield improvement under DDSR. High-throughput, timely, cost-effective, and easy-to-use genotyping technologies offer modern tools and techniques to the plant breeding community to be used for genomics-assisted breeding^18^. With the advent of whole-genome sequencing, high-density SNP arrays enable the detection of significant marker-trait associations, quantitative trait loci (QTLs), and candidate genes in genome-wide association studies (GWAS)^19,20^. High-density SNP arrays covering the whole genome are necessary to monitor the recombination breakpoints in the diverse panel^20,21^. GWAS can be used to identify the specific functional genetic variants such as alleles and QTLs that are linked to the phenotypic differences in a particular trait of interest to facilitate the detection of traits and selection of genotypes possessing traits of interest^22^. Association studies involving landraces, diverse germplasm, multi-parent breeding populations, NAM (nested association mapping) populations^23–26^, and breeding populations^27^ have identified significant marker-trait associations and fine-mapped candidate genes^28^ that will help rice breeders to undertake a systematic breeding program. GWAS have been reported as a major success in different crop species, including maize^29,30^, soybean^31^, foxtail millet^32^, rice^24^, chickpea^33^, and pigeonpea^34,35^. The proper understanding of the traits, donors, and markers underlying the genes needed for grain yield improvement and root traits enhancing nutrient uptake under DDSR may lead to breakthroughs in developing climate-resilient DDSR-adapted rice varieties.

In the present study, GWAS were conducted on 39 traits, including seedling establishment, root morphological, plant morphological, grain yield, and yield-contributing traits, in a complex mapping population. The aim of the study is to identify significant associations and QTLs/putative candidate genes to be used directly in marker-assisted breeding programs to develop high-yielding and nutrient-efficient rice varieties for DDSR conditions.

## Results

### Phenotypic variations of targeted traits

More variability was observed in the dry season (DS) than in the wet season (WS) under DDSR. No significant variability was observed for relative growth rate from 22 to 29 days after seeding (DAS) and from 15 to 29 DAS, maximum root length at any sampling point, flag-leaf length and width, leaf color chart (LCC), and total biomass at flowering in 2015WS (Table 1). Significant variability was observed for all the observed traits in 2016DS except for root length at 29 DAS and vegetative vigor (Table 1). The mean grain yield of the complex mapping population ranged from 3020 kg ha^−1^ in 2015WS to 4263 kg ha^−1^ in 2016DS. Negative skewness was observed for days to 50% flowering, grain yield (2016DS), SPAD (2016DS), and panicle length. Positive skewness was observed for days to first and full emergence, flag-leaf angle, flag-leaf area, nodal roots, root hair length, root hair density, total number of productive tillers, 1000-grain weight, plant height, and culm diameter. The parents UPLRi 7 and IR 74371-70-1-1 showed a better performance in terms of grain yield and number of nodal roots in both the wet and dry season (Table 1). The parent IRRI 123 had better root hair length and density in 2016DS. The parents UPLRi 7 and IRRI 148 had more flag-leaf area than the other parents. In terms of SPAD and LCC, the parents IR 74371-70-1-1 and UPLRi 7 showed a better performance. Across seasons, UPLRi 7 and Vandana showed better lodging resistance than the other parents.

**Table 1:**
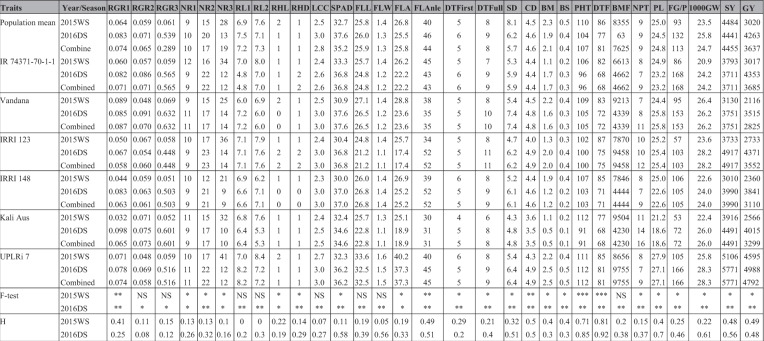
Mean data of the co mplex mapping population and six parents for different seedling-establishment, root, plant morphological, yield, and yield-contributing traits under dry direct-seeded conditions.

RGR1: relative growth rate from 15 to 22 DAS, RGR2: relative growth rate from 22 to 29 DAS, RGR3: relative growth rate from 15 to 29 DAS, NR1: number of nodal roots at 15 DAS, NR2: number of nodal roots at 22 DAS, NR3: number of nodal roots at 29 DAS, RL1: maximum root length (cm) at 15 DAS, RL2: maximum root length (cm) at 22 DAS, RHL: root hair length, RHD: root hair density, LCC: leaf color chart, SPAD: chlorophyll content, FLL: flag-leaf length, FLW: flag-leaf width, FLA: flag-leaf area, FLAngle: flag-leaf angle, DTFirst: days to first emergence, DTFull: days to full emergence, SD: stem diameter (mm), CD: culm diameter (mm), BS: bending strength (kg cm), BM: bending moment (kg cm^2^), PHT: plant height (cm), DTF: days to 50% flowering (days), BMF: biomass at 50% flowering (g), NPT: number of productive tillers, PL: panicle length (cm), NFG/P: number of filled grains/panicle, 1000 GW: 1000-grain weight (g), SY: straw yield (kg ha^−1^), GY: grain yield (kg ha^−1^), H: broad-sense heritability.

NS: non-significant, *significant at <0.05 level, **significant at <0.01 level, *significant at <0.001 level.

**Figure 1:**
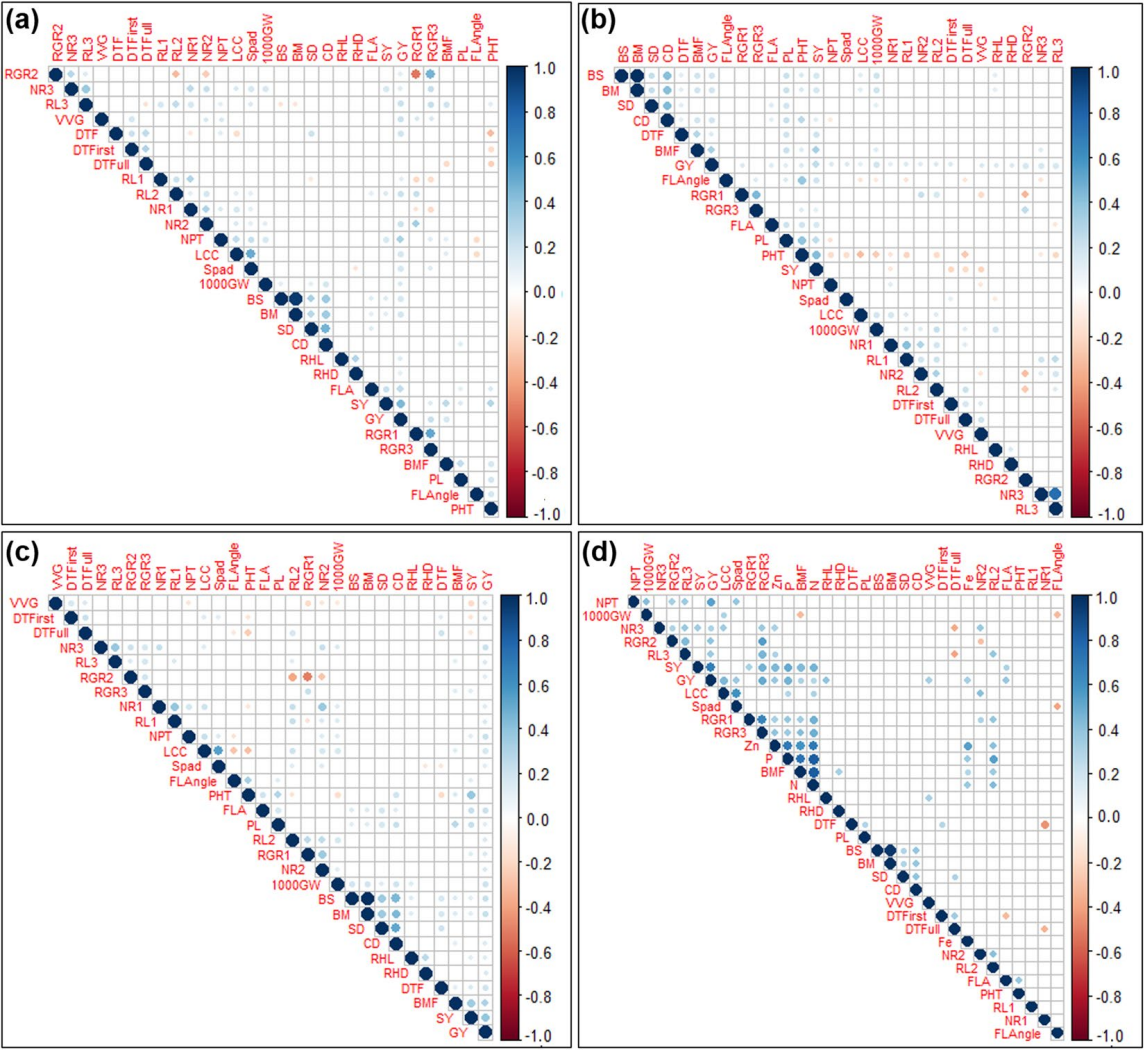
Phenotype-phenotype correlation plot of different seedling establishment, root, grain, and grain yield-contributing traits considering whole population in (**a**) 2015WS, (**b**) 2016DS, (**c**) combined seasons, and (**d**) different seedling establishment, root, nutrient uptake, grain and grain yield-contributing traits considering 60 progenies (30 high-yielding and 30 low-yielding used for nutrient uptake analysis). Blue color indicates significant positive correlation and red color indicates significant negative correlation among different traits. *RGR1: relative growth rate from 15 to 22 DAS, RGR2: relative growth rate from 22 to 29 DAS, RGR3: relative growth rate from 15 to 29 DAS, NR1: number of nodal roots at 15 DAS, NR2: number of nodal roots at 22 DAS, NR3: number of nodal roots at 29 DAS, RL1: maximum root length (cm) at 15 DAS, RL2: maximum root length (cm) at 22 DAS, RL3: maximum root length (cm) at 29 DAS, RHL: root hair length, RHD: root hair density, LCC: leaf color chart, SPAD: chlorophyll content, FLL: flag-leaf length, FLW: flag-leaf width, FLA: flag-leaf area, FLAngle: flag-leaf angle, DTFirst: days to first emergence, DTFull: days to full emergence, SD: stem diameter, CD: culm diameter, BS: bending strength (kg cm), BM: bending moment (kg cm*^2^*), PHT: plant height (cm), DTF: days to 50% flowering (days), BMF: biomass at 50% flowering (g), VVG: vegetative vigor score, NPT: number of productive tillers, PL: panilcle length (cm), NFG/P: number of filled grains/panicle, 1000 GW: 1000-grain weight (g), SY: straw yield (kg ha*^−*1*^*), GY: grain yield (kg ha*^−*1*^*), N: nitrogen, P: phosphorus, Fe: iron, Zn: zinc*.

Grain yield was significantly and positively correlated with seedling-establishment traits (days to first emergence, vegetative vigor), root- and nutrient uptake-related traits (nodal roots, root length, root hair length, leaf color chart, SPAD), lodging-resistance traits (bending strength, bending moment, culm diameter), and grain yield-contributing traits (number of productive tillers, filled grains per panicle, 1000-grain weight, biomass at 50% flowering, straw yield) in 2015WS (Fig. 1a), in 2016DS (Fig. 1b), and in combined seasons analysis (Fig. 1c). Nutrient (nitrogen, phosphorus, iron, zinc) uptake was significantly and positively correlated with nodal roots, root length, root hair length, LCC, SPAD, number of productive tillers, biomass at 50% flowering, straw yield, and grain yield (Fig. 1d).

### Population structure and linkage disequilibrium (LD)

**Figure 2:**
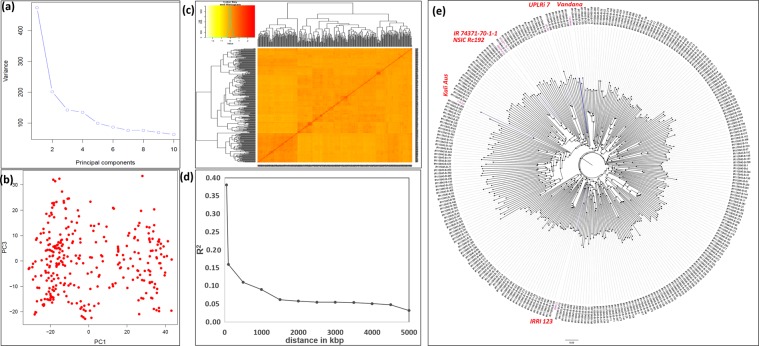
Genetic relatedness and population structure of complex mapping breeding population association panel: (**a**) a screen plot showing most of the variability explained by the first two principal components (PCs) for association study, (**b**) variation of the first two PCs, (**c**) kinship values, (**d**) average LD as a function of inter-SNP marker distance estimated in the complex mapping breeding population association panel, and (**e**) phylogenetic neighbor-joining tree of the complex mapping population used for phenotyping under direct-seeded conditions.

Population structure and LD decay were estimated using the genetic structure of the 300 progenies from the complex mapping population and 6 parents using 10,588 SNPs distributed across all 12 rice chromosomes. The GAPIT screen plot showed that the first two principal components (PCs) were informative, and then a gradual decrease occurred (Fig. 2a) until the tenth PC. The first PC explained 38.3% and the second PC explained 13.2% of the total variance (Fig. 2b). All the progenies were divided into two distinct major groups and further subdivided into subgroups (Fig. 2c). The kinship heatmap showed that most of the kinship value was concentrated from the *0.0 to 0.5 level (0.0: no relatedness, 0.5: weak relatedness), indicating a weak relatedness in the association panel (Fig. 2c). Pairwise linkage disequilibrium (r^2^) was calculated between all 10,588 SNPs. The average r^2^ for the closest markers of 5 kb started at 0.38 and the LD (r^2^) dropped to approximately half of its maximum value (0.16) at around 100 kb (Fig. 2d). The phylogenetic structure of the complex mapping population panel was illustrated using the unweighted NJ tree (Fig. 2e).

### Significant MTAs for molecular breeding for DDSR

A total of 971,790 SNPs were called from the genotyping by sequencing (GBS) data. A stringent selection criterion to filter out the SNPs was used, including missing percentage, MAF (minor allele frequency), and percent heterozygosity, to select the panel of robust SNPs. After filtering the SNPs with call rate 85% and MAF of >5%, a total of 10,588 polymorphic SNPs were retained to conduct GWAS studies on 39 traits measured in 2015WS and 2016DS. The significant MTAs that surpass the Bonferroni threshold and that were consistent over seasons and combined seasons analysis were reported in the present study. The marker-trait associations identified on the same chromosome within a region of 2.5 Mb were assigned as putative QTLs.

**Figure 3:**
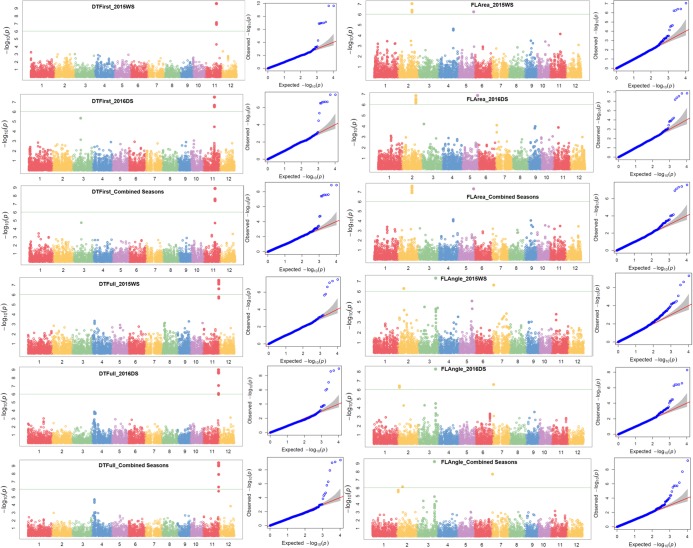
Manhattan and Q-Q plots of genome-wide association mapping of seedling-establishment traits (DTFirst: days to first emergence, DTFull: days to full emergence) and plant morphological traits (FLA: flag-leaf area, FLAngle: flag-leaf angle) across seasons under direct-seeded cultivation conditions. The y axis in each graph represents −log_10_P for the p-value of the MTAs, while linkage groups are indicated on the x axis.

**Figure 4:**
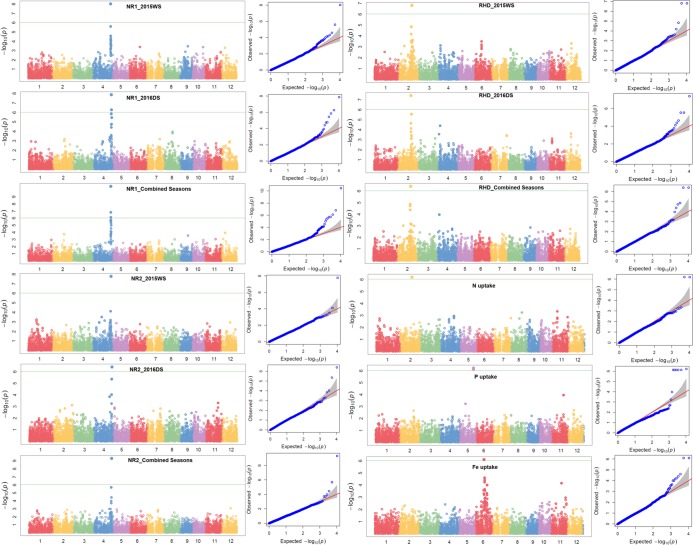
Manhattan and Q-Q plots of genome-wide association mapping of root traits (NR1: nodal roots at 15 days after seeding, NR2: nodal roots at 22 DAS, RHD: root hair density) across seasons and nutrient uptake (N: nitrogen, P: phosphorus, Zn: zinc) under direct-seeded cultivation conditions. The y axis in each graph represents −log_10_P for the p-value of the MTAs, while linkage groups are indicated on the x axis.

**Figure 5:**
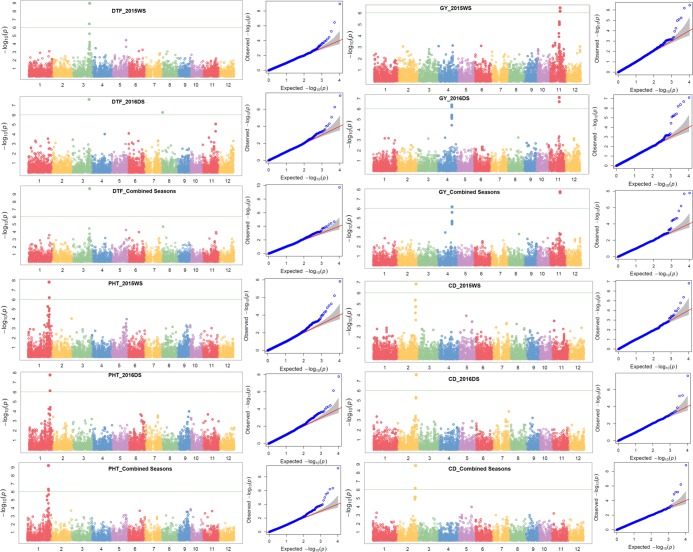
Manhattan and Q-Q plots of genome-wide association mapping of agronomic traits (DTF: days to 50% flowering, PHT: plant height, GY: grain yield) and lodging-resistance traits (CD: culm diameter) across seasons under direct-seeded cultivation conditions. The y axis in each graph represents −log_10_P for the p-value of the MTAs, while linkage groups are indicated on the x axis.

A total of 10 significant trait-SNP associations and 25 QTLs associated with 25 traits of interest were identified. The QTL span ranged from 141 to 2345 kb. The false discovery rate (FDR) ranged from 0.0227 to 1.21 × 10^−6^, 0.192 to 2.44 × 10^−6^, and 0.190 to 3.66 × 10^−7^ in 2015WS, 2016DS, and combined seasons analysis, respectively (Table 2). Detailed information on SNPs, SNP position, QTL span, p-values, R^2^, and FDR is shown in Table 2. The Manhattan plots depicting -log (p-values) and Q-Q plots (quantile quantile) showing the expected vs observed p-values for the MTAs are presented in Fig. 3 (seedling-establishment traits and plant morphological traits), Fig. 4 (root traits and nutrient uptake), and Fig. 5 (agronomic traits, grain yield, and lodging-resistance traits).

**Table 2:** Identified significant SNP-trait associations in genome-wide study on the complex mapping population.

Trait	SNP	Chr	Position	QTL span (kb)	2015WS			2016DS			Source: http://qtaro.abr.affrc.go.jp	
					p-value	R^2^ (%)	FDR	p-value	R^2^ (%)	FDR	Genes	QTLs
RGR1	S1_37335658	1	37335658, 37335658–37605610	270	—	—	—	3.42 × 10^−9^	13	3.61 × 10^−5^	*Lax*^39^, *moc2*^40^	—
RGR2	S1_37542157	1	37542157, 37542133–37542192	0.06^a^	—	—	—	7.13 × 10^−9^	13	7.52 × 10^−6^	*Lax*^39^, *moc2*^40^	—
RGR3	S1_42364262	1	42364262, 42364257–42906879	543	—	—	—	2.31 × 10^−10^	16	2.44 × 10^−6^	—	—
NR1	S4_31903323	4	31903323–32251982	349	9.29 × 10^−9^	13	9.81 × 10^−5^	1.35 × 10^−8^	19	0.000143	*nal1*(*nal5*)^41^	*rfw4a*^44^, root thickness^42,43^, rooting depth^42^
NR2	S4_32664173	4	32664173, 31903323–32664173	761	1.70 × 10^−8^	16	0.000179	3.95 × 10^−7^	13	0.004172	*nal1*(*nal5*)^41^	*rfw4a*^44^, root thickness^42,43^, rooting depth^42^
RHL	S5_18574046	5	18574046, 18574046–18574116	0.07^a^	7.88 × 10^−10^	13	2.59 × 10^−6^	3.99 × 10^−7^	10	0.001053	*OsIPT3* ^45^	—
RHD	S2_24278920	2	24278920, 23246555–24278925	1032	1.55 × 10^−7^	10	0.000818	2.84 × 10^−6^	8	0.00998	*EL5* ^46^ *, OsCKI1* ^47^ *, OsNAR2.1* ^48^	—
FLA	S2_24874074	2	24874074, 24874069–24874083	0.014^a^	9.22 × 10^−8^	13	0.000973	2.78 × 10^−7^	12	0.000978	—	—
	S5_27946781	5	27946781	—	5.52 × 10^−6^	11	0.001283	1.35 × 10^−7^	13	0.000739	—	—
FLAngle	S3_30920007	3	30920007	—	4.93 × 10^−8^	13	0.000520	5.12 × 10^−9^	19	5.40 × 10^−5^	*OSH1*^72^, *OsGRAS19*^61^	—
	S7_859891	7	859891	—	2.33 × 10^−7^	12	0.001231	2.65 × 10^−7^	17	0.000817	—	—
	S2_10388315	2	10388315	—	5.05 × 10^−7^	11	0.001775	—	—	—	—	—
	S2_2772428	2	2772428, 2772428–2772461	0.033^a^	—	—	—	3.87 × 10^−7^	16	0.000817	—	—
	S5_23310970	5	23310970	—	8.17 × 10^−6^	9	0.021549	—	—	—	—	—
DTFirst	S11_19839428	11	19839428, 19839399–19839440	0.041^a^	2.37 × 10^−10^	21	1.25 × 10^−6^	3.20 × 10^−8^	15	0.000169	—	*qGP-11*, *qGI-11*^53^, *yld11.1*, *gpl11.1*, *gw11.1*^54^
DTFull	S11_28121354	11	28121354, 28121310–28121357	0.047^a^	3.36 × 10^−8^	19	0.000265	1.02 × 10^−9^	22	8.79 × 10^−6^	—	—
SPAD	S11_1639964	11	1639964, 1639964–1639983	0.019^a^	6.31 × 10^−8^	13	0.000665	1.55 × 10^−7^	12	0.001633	*OsNAC10* ^73^	—
	S2_5964826	2	5964826		4.61 × 10^−7^	12	0.001621	6.70 × 10^−7^	11	0.002359	*CYP734A2*^74^, *SPK2*(*SYG2*)^49^	—
	S3_9581723	3	9581723	—	—	—	—	7.28 × 10^−5^	7	0.19204		—
BS	S3_18257114	3	18257114, 18257114–18257152	0.038^a^	3.75 × 10^−8^	19	0.000396	—	—	—	*cwa1/bc1* ^75^	—
BM	S3_18257114	3	18257114, 18257114–18257152	0.038^a^	2.35 × 10^−8^	18	0.000248	—	—	—	*cwa1/bc1* ^75^	—
CD	S2_30984762	2	30984762, 30984762–32312089	1327	4.30 × 10^−6^	10	0.022673	—	—	—	*bc3* ^50^	—
SD	S3_28113794	3	28113794, 27926957–28113794	187	3.54 × 10^−8^	14	0.000187	1.10 × 10^−7^	13	0.000765	*SCM3* ^5^	—
1000 GW	S10_11387999	10	11387999, 11387999–11388014	0.015^a^	1.92 × 10^−7^	14	0.000682	1.37 × 10^−7^	23	0.000721	—	*ssd10*^76^, *qSPBp10-2*^77^
	S11_27252113	11	27252113	—	1.87 × 10^−7^	14	0.000682	2.13 × 10^−7^	22	0.000749	—	—
PL	S1_14102690	1	14102690, 13961627–14102698	141	2.96 × 10^−10^	18	1.21 × 10^−6^	3.44 × 10^−7^	16	0.000839	—	—
	S11_27831001	11	27831001	—	—	—	—	2.97 × 10^−7^	16	0.000839	—	—
NPT	S11_4326232	11	4326232, 4326232–4326316	0.084^a^	1.75 × 10^−7^	17	0.000927	6.83 × 10^−8^	15	0.00024	—	—
PHT	S1_38286772	1	38286772, 35953453–38286810	2333	1.46 × 10^−8^	28	0.000154	1.68 × 10^−8^	39	0.000177	*Sd1*^51^, *qDTY*_*1.1*_^52^	—
DTF	S3_31345368	3	31345368, 30831775–31345368	514	1.09 × 10^−9^	29	1.15 × 10^−5^	2.37 × 10^−8^	23	0.00025	*MADS14* ^78^ *, pap2* ^79^ *, osmads34* ^80^	—
	S8_2061021	8	2061021	—	—	—	—	5.35 × 10^−7^	21	0.002822	—	—
GY	S11_18903704	11	18903704, 16558222–18903704	2345	3.44 × 10^−7^	22	0.003506	2.00 × 10^−7^	21	0.001057	—	*qGP-11, qGI-11* ^53^ *, yld11.1, gpl11.1, gw11.1* ^54^
	S4_25915108	4	25915108, 25915108–25915179	0.071^a^	—	—	—	4.65 × 10^−6^	19	0.008182	—	—
Fe uptake	S6_14891911	6	14891911–14891917	0.006^a^	7.96 × 10^−7^	79	0.004214	—	—	—	*OsPT9*, *OsPT10*^55^, *OsGLK1*^56^, *nyc3*^57^	—
N uptake	S2_20846594	2	20846594–20846633	0.039^a^	6.43 × 10^−7^	84	0.003402	—	—	—	*DP2*^58^, *RCN2*^59^, *OsAPX8*^60^, *OsNAR2.1*^*48*^	—
P uptake	S5_24675892	5	24675892, 24675892–24675951	0.059^a^	5.91 × 10^−7^	83	0.001308	—	—	—	*OsRPK1*^81^, *OsCCaMK*^62^, *OsHAP3B*^63^; *OsTPS1*^63^, *OsSTN8*^64^	—

RGR1: relative growth rate from 15 to 22 DAS, RGR2: relative growth rate from 22 to 29 DAS, RGR3: relative growth rate from 15 to 29 DAS, NR1: number of nodal roots at 15 DAS, NR2: number of nodal roots at 22 DAS, RHL: root hair length, RHD: root hair density, SPAD: chlorophyll content, FLA: flag-leaf area, FLAngle: flag-leaf angle, DTFirst: days to first emergence, DTFull: days to full emergence, SD: stem diameter, CD: culm diameter, BS: bending strength, BM: bending moment, 1000 GW: 1000-grain weight (g), PL: panicle length, NPT: number of productive tillers, PHT: plant height, DTF: days to 50% flowering, GY: grain yield, Fe uptake: iron uptake, N uptake: nitrogen uptake, P uptake: phosphorus uptake, DS: dry season, WS: wet season, R^2^: percent phenotypic variance explained by SNP, FDR: false discovery rate. ^a^The associated SNPs with very small intervals (≤14 kb) can be considered as the same locus.

A total of eight QTLs on chromosomes 2, 4, 5, and 6 were reported to be associated with root morphological traits and nutrient uptake (N, P, Fe). Putative QTLs were found for nodal root number on chromosome 4 spanning the 760 kb region, for root hair length spanning the 0.07 kb region on chromosome 5, and for root hair density spanning the 1032 kb region on chromosome 2. A 3.4 Mb region on chromosome 2 was associated with QTLs for root hair density and nitrogen uptake. The QTLs for root hair length and phosphorus uptake were 4.6 Mb apart from each other on chromosome 5. On the long arm of chromosome 3, a 3.4 Mb region was identified to be associated with flag-leaf angle, days to 50% flowering, and stem diameter. QTLs were found for lodging resistance traits such as stem diameter spanning the 187 Mb region on chromosome 3, for bending strength and bending moment spanning the 0.038 kb region on chromosome 3, and for culm diameter spanning 1.3 Mb on chromosome 2. A 9 Mb region on the long arm of chromosome 5 was reported to be associated with traits such as root hair length, phosphorus uptake, and flag-leaf area. The 6.9 Mb region on the long arm of chromosome 1 was reported to be associated with plant height and relative growth rate. The grain yield QTL harbors a 2.3 Mb region on chromosome 11. The long arm of chromosome 11 was reported to be associated with grain yield, grain yield-contributing traits (panicle length, 1000-grain weight), and seedling-establishment traits (days to first and full emergence) under DDSR. The QTLs for days to first emergence and grain yield were separated from each other by a 0.9 Mb region on chromosome 11. In addition to the grain yield QTL on chromosome 11, another grain yield QTL on chromosome 4 in 2016DS and combined seasons analysis was observed. Over the seasons, the p-value of the SNPs that passed the Bonferroni threshold ranged from 0.00032 to 4.48 × 10^−8^ for nodal roots at 15 DAS, 0.000208 to 2.00 × 10^−10^ for nodal roots at 22 DAS, 0.05 to 4.96 × 10^−11^ for grain yield, and 2.933 × 10^−7^ to 3.207 × 10^−7^ for P uptake. The SNPs S3_18257114 and S3_18257152 on chromosome 3 were observed to be significantly associated with bending strength and bending moment both and S4_31903323 on chromosome 4 with nodal root number at different growth points. The associated SNPs with a very small interval (≤14 kb) can be considered as the same locus.

**Figure 6:**
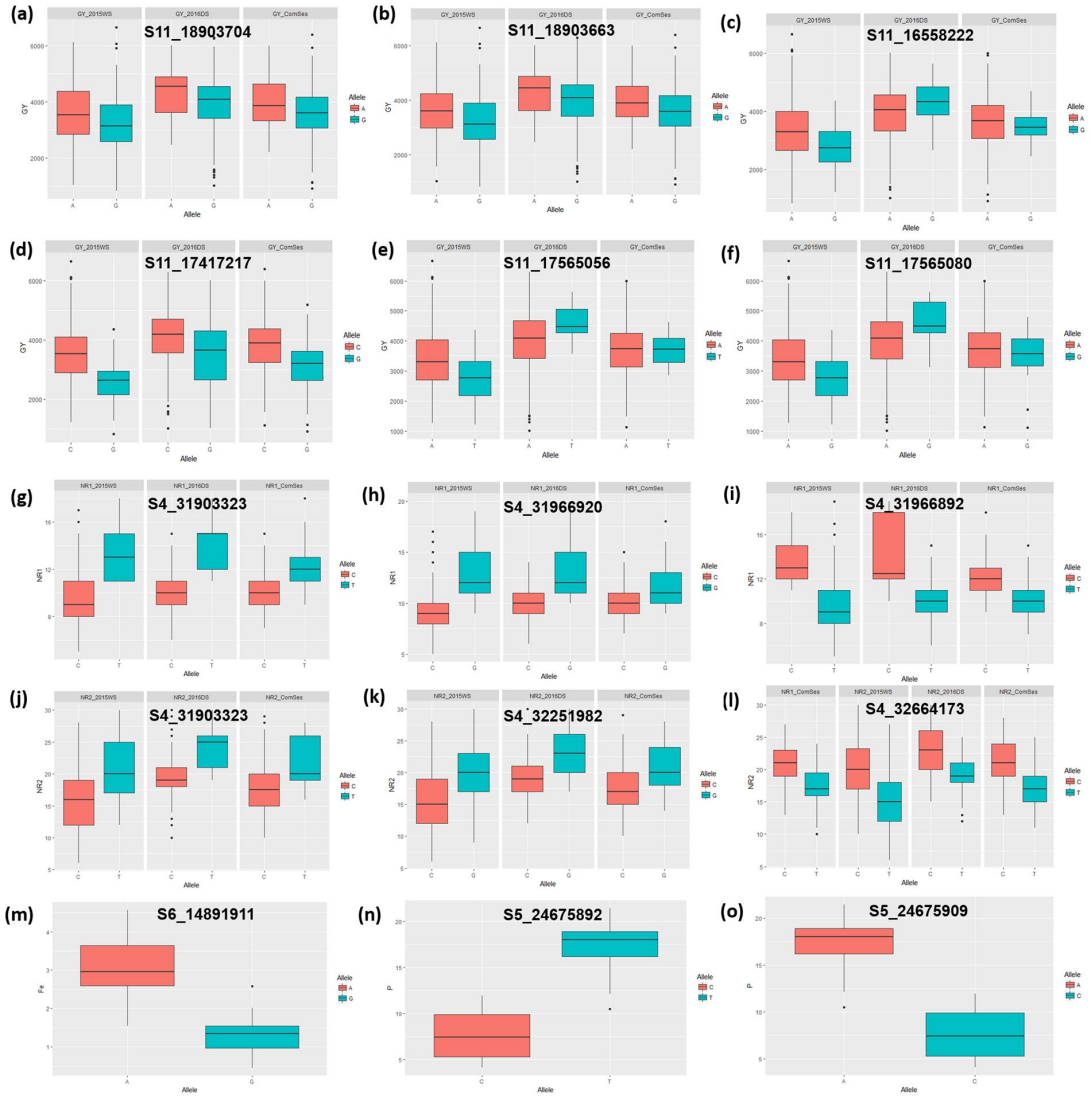
Comparison of allelic effects for marker-trait association for (**a–f**) grain yield, (**g–i**) root traits [NR1: nodal roots at 15 DAS], (**j–l**) root traits [NR2: nodal roots at 22 DAS] (**m**), iron [Fe] uptake (**n,o**), and P [phosphorus] uptake. The significant difference between the mean values of the allelic class was determined using the Kruskal–Wallis test.

The allelic effects of the significantly associated markers for root, nutrient uptake, and grain yield traits are shown in Fig. 6. The detailed description of the identified candidate genes underlying the significant marker-trait association/putative QTLs is presented in Supplementary Table S1. The identified candidate genes in the present study that were in the vicinity of the previously reported SNPs/putative QTLs warrant further investigation and validation.

### Performance of selected promising progenies

Eight improved breeding progenies possessing better root morphology for higher nutrient uptake and higher yield under DDSR were identified (Table 3). The percentage improvement in the promising breeding progenies ranged from 17% to 52% for nitrogen uptake over the best performing parent, Vandana. The percentage improvement for phosphorus and iron uptake in the selected progenies was 17% to 69% and 14%, respectively, over the parent Kali Aus (Table 3). The best performing progenies for zinc uptake showed percentage improvement ranging from 13% to 97% over the best parent identified for zinc uptake (IR 74371-70-1-1) under DDSR. The percentage improvement of number of nodal roots at 29 DAS in selected promising progenies varied from 4% to 92%, 7% to 14%, and 5% to 63% in 2015WS, 2016DS, and combined seasons, respectively, over parent Vandana (Table 3). The percentage grain yield advantage of the promising progenies ranged from 0.6% to 45% in 2015WS, 0.6% to 6% in 2016DS, and 0.1% to 18% in combined seasons analysis over the best yielding parent (UPLRi 7) across seasons (Table 3).

**Table 3:** Significant variation (t-test) for selected promising progenies for nutrient uptake, root traits, and grain yield under dry direct-seeded conditions.

Progeny	N uptake	P uptake	Fe uptake	Zn uptake	NR3			RHL			RHD			GY (kg ha^−1^)		
					2015 WS	2016 DS	Comb seas	2015 WS	2016 DS	Comb seas	2015 WS	2016 DS	Comb seas	2015 WS	2016 DS	Comb seas
IR 115845-B-310-B	75.3	18.1	2.6	0.353	33	16	25	2	1	1	2	0	1	5046	4105	4575
IR 115845-B-36-B	61.9	10.6	1.8	0.166	48	15	31	1	1	1	2	1	1	4759	5275	5017
IR 115845-B-61-B	76.8	17.9	4.6	0.391	33	15	24	1	1	1	2	1	2	4495	4603	4549
IR 115845-B-286-B	59.7	10.4	2.5	0.262	42	14	28	2	1	2	2	1	2	4761	5019	4690
IR 115845-B-398-B	67.7	14.9	1.7	0.172	32	14	23	2	1	2	2	1	1	4621	4747	4684
IR 115845-B-435-B	65.9	15.8	2.4	0.147	27	16	22	1	1	1	1	1	1	5029	4289	4659
IR 115845-B-154-B	90.5	21.4	1.6	0.230	30	13	22	1	1	1	2	2	2	6656	4623	5640
IR 115845-B-419-B	68.5	18.0	2.3	0.225	26	13	20	1	1	1	1	1	1	4874	4721	4797
IR 74371-70-1-1^+^	46.4	8.2	1.5	0.199	34	12	23	1	1	1	1	2	2	3017	4353	3685
Vandana^+^	69.7	12.4	3.3	0.184	25	14	19	2	0	1	1	1	1	2116	3515	2815
IRRI 123^+^	60.8	10.9	1.5	0.188	30	14	25	1	2	1	1	2	1	2733	4371	3552
IRRI 148^+^	56.4	10.2	2.0	0.127	21	9	15	1	0	1	1	—	1	2360	3841	3101
Kali Aus^+^	57.8	12.7	4.0	0.167	32	10	21	1	1	1	1	1	1	2566	4015	3291
UPLRi 7^+^	44.0	8.8	1.4	0.185	33	12	23	2	1	2	1	—	1	4595	4988	4792
IR 94225-B-82-B	55.5	11.1	2.2	0.129	28	16	22	1	1	1	1	1	1	2132	2668	2400
IR 94226-B-177-B	55.2	11.3	2.0	0.150	33	12	23	2	1	2	2	1	1	2238	3867	3053
IR 91648-B-32-B-B	74.3	26.3	2.1	0.215	22	10	16	2	1	1	1	1	1	2753	2104	2428
Mean	66.1	14.5	2.3	0.2	31	13	22	1	1	1	1	1	1	3837	4227	4008
SE mean	2.86	1.02	0.18	0.01	1.47	0.36	0.86	0.1	0.1	0.07	0.08	0.1	0.06	272	178	188
t-test	23.1**	14.2**	12.5**	13.9**	20.8**	35.3**	24.8**	15.4**	10.9**	17.5**	17.8**	11.5**	19.4**	14.1**	23.8**	21.3**

^+^Parent, DS: dry season, WS: wet season, Comb seas: combined seasons, **significance at <0.01 level, NR3: number of nodal roots at 29 DAS, RHL: root hair length, RHD: root hair density, GY: grain yield (kg ha^−1^), N uptake: nitrogen uptake (kg ha^−1^), P uptake: phosphorus uptake (kg ha^−1^), Fe uptake: iron uptake (kg ha^−1^), Zn uptake: zinc uptake (kg ha^−1^).

## Discussion

### Phenotypic variability and correlation studies

Higher nutrient uptake, weed competitiveness, good crop stand, yield improvement, and adaptability are some of the prerequisites to developing resource-efficient high-yielding rice varieties for dry direct-seeded conditions^36^. The significant differences for different traits in the progenies indicated the existence of variability in the population that can be exploited for marker-assisted breeding. The fast and uniform seedling establishment represented by more nodal roots, longer root length, and more root hair length and density would be important traits for DDSR. The similarity in LCC and SPAD values across the progenies indicated the LCC as a cheap alternative to SPAD, which is expensive for farmers to buy to decide on the time and dose of fertilizer application under DDSR.

The significant positive correlation of grain yield with yield-contributing traits and seedling-establishment traits indicated the role of seedling- and reproductive-stage traits in improving grain yield. The significant positive correlation of nutrient uptake with root traits and plant morphological traits such as flag-leaf length, width, and area and chlorophyll content (which was indirectly estimated with LCC and SPAD) indicated the role of these traits in enhancing photosynthetic ability. Further, the preferential distribution of photosynthetic assimilates from leaves to vigorous roots may allow rice to uptake nutrient efficiently under DDSR.

The direction of the correlation (whether positive or negative) across seasons was the same but the significance levels were different. Traits such as DTFirst emergence, VVG, NR, RHD, RHL, LCC, CD, and PHT showed the effect of seasonal variability. Early vigor and uniform emergence are complex traits that are influenced by different environmental and soil factors. The seasonal variation also indicated the role of phenotypic plasticity behavior of different traits in increasing yield across different seasons. A better understanding of the relationship between root traits, plant morphological traits, and grain yield across different seasons, soil profiles, and developmental stages with water-nutrient uptake is likely to provide a novel dimension in selecting traits for developing the high-yielding varieties needed for DDSR. The correlation among different root traits and nutrient uptake indicated the complementary functional roles of root traits for the acquisition of different soil resources and improving grain yield and adaptability under DDSR.

### Population structure analysis

Population stratification and uneven distribution of alleles can result in non-functional and spurious associations. In the present study, the kinship value was concentrated from *0.0 to 0.5, indicating weak relatedness in the GWAS panel. Significant variability has been observed for all the measured phenotypic traits in the present study. The *aus* and *indica* subpopulations used in developing the present population harbor alleles for different traits of interest and are reported to show adaptability to different environments and geographies. The *aus* subpopulation carries alleles for root traits and other traits contributing to drought tolerance while the *indica* subpopulation harbors huge genetic variation.

### Marker-trait associations

To take advantage of existing variations^37^, with a high-throughput phenotyping-genotyping platform, advanced statistical strategies^25,26,38^, marker-trait associations, and/or GS (genomic selection)^27^, association mapping was performed directly on the complex mapping population in the present study.

The significant phenotypic variability present in the complex mapping population and the high genome coverage provided a strong base for the genome-wide association study on the complex mapping population. The allelic distribution of each progeny in the population presented an approximately 25% contribution of Vandana, 25% of NSICRc 192, and 12.5% each of the other four parents (IR 74371-70-1-1, Kali Aus, IRRI 123, and UPLRi 7). Among the 25 identified QTLs, 16 were located in the proximity of earlier identified candidate genes (http://qtaro.abr.affrc.go.jp), and, among the 10 identified significant marker-trait associations, two were located in the proximity of earlier identified candidate genes (http://qtaro.abr.affrc.go.jp) (Supplementary Table S1). A significant association signal on chromosome 1 was detected for relative growth rate at different time points in 2016DS. The reported region in the present study was located in the promixity of *lax*^39^ and *moc2*^40^ candidate genes controlling shoot branching and tiller growth, respectively, indicating the robustness of the associations identified in the present study.

For the number of nodal roots at 15 (NR1) and 22 DAS (NR2) on chromosome 4, ~290 kb upstream, a positional match regulating leaf and adventitious root development in rice was identified as the *nal1* (*nal5*) gene, NARROW LEAF1^41^. he QTLs for nodal roots identified in the present study colocating with the QTLs for root thickness^42,43^ and rooting depth^42^ and in the region of *rfw4a*, a QTL was identified for root fresh weight^44^, indicating the role of the region (30–33 Mb) on the long arm of chromosome 4 in root development. The QTL for root hair length on chromosome 5 was observed to be colocated with the earlier identified gene *OsIPT3*, a gene tightly linked with root growth in rice^45^, and with *qRHD*_*5.1*_, a QTL for root hair density^4^. The root hair density QTL on chromosome 2 was found to be colocated with the earlier reported genes *EL5*^46^, *OsCK11*^47^, and *OsNAR2.1*^48^, which had shown a major role in root development and nitrate uptake, respectively. This QTL is also reported to be colocated with the QTL for nitrogen uptake in the present study. This may explain the role of root hair density in the uptake of the nitrate form of nitrogen under DDSR. The SNP S2_5964826 on chromosome 2 associated with SPAD was identified to be colocated with *SPK2* (*SYG2*), a gene that inhibits phosphate starvation responses in rice^49^.

The earlier reported gene *bc3* (Rice BRITTLE CULM 3) that encodes *OsDRP2B*, a classical dynamin that is essential for secondary cell wall synthesis^50^, is located in the region of the QTL identified for culm diameter on chromosome 3 (30.98–32.31 Mb) in the present study. The QTL for stem diameter on chromosome 3 was found to be colocated with the earlier reported QTL/gene *SCM3*^5^. In the present study, the QTL for plant height on chromosome 1 colocalized with the rice semi-dwarfing gene^51,52^ (*Sd1)* encoding the mutant enzyme gibberellin 20-oxidase that is involved in the synthesis of gibberellin and with another QTL, *qPHT*_*1.1*_, reported by Sandhu *et al*.^4^. The 2.3 Mb region on the long arm of chromosome 11 was reported to be significantly associated with grain yield. This 2.3 Mb region was localized in the promixity of earlier identified QTLs^53,54^ (*qGP-11*, *qGI-11* and *yld11.1*, *gpl11.1*, *gw11*) that were involved in improving grain yield in an *Oryza sativa* × *O. rufipogon* BC_2_F_2_ population under upland conditions. An additional QTL for grain yield under DDSR was reported on chromosome 4 in 2016DS and combined seasons analysis. Interestingly, the QTL on chromosome 6 for iron uptake under DDSR in the present study was located near the earlier reported genes *OsPT9*^55^ (involved in phosphorus uptake in rice), *OsGLK1*^56^ (induced chloroplast development in rice), and *nyc3*^57^ (stay green phenotype during leaf senescence in rice). The genomic region from 20 to 24 Mb on chromosome 2 was reported to be associated with genes *DP2*^58^ (dense panicle), *RCN2*^59^ (panicle morphology), *OsAPX8*^60^ (photosynthesis and adaptation under photo-oxidative stress), and *OsNAR2.1*^48^ (nitrate uptake transporter). This signified that the QTL for nitrogen uptake underlying the genetic region is associated with source and sink capacities in rice.

The phosphorus uptake QTL on chromosome 5 was located in the region associated with the *OsRPK1* gene that regulates root development^61^, *OsCCaMK* gene for microbial symbiosis^62^, *OsHAP3B* and *OsTPS1* genes for chloroplast biogenesis^63^, and *OsSTN8* gene for protein phosphorylation of photosystem II^64^. The colocation of identified QTLs or significant SNPs with earlier reported genes/QTLs for root development, photosynthetic traits, nutrient transporter, nutrient uptake, and stress-responsive genes further confirms the contribution of these traits/QTLs in enhancing yield and adaptability under DDSR. The identification of novel markers/SNPs/candidate genes in the QTL region helps to harness their benefits in genomics-assisted selection.

### GWAS: traits and progenies

The 10 identified significant MTAs, 25 QTLs associated with 25 traits of interest, and the 8 selected promising breeding progenies with favorable allele combinations can be deployed after validation for improving grain yield and adaptatbility using molecular breeding. The parental alleles providing improved grain yield under DDSR were contributed by parents Kali Aus, UPLRi 7, IR 74371-70-1-1, and IRRI 123. The parental alleles reported to be reponsible for the increase in nodal root number were contributed by Kali Aus and for nutrient uptake by the parents (including Kali Aus, IRRI 148, IRRI 123, and Vandana). In the identification of suitable traits needed for DDSR, promising progenies possessing those traits and the SNPs associated with colocated traits would significantly promote genomics-assisted breeding in developing DDSR varieties. The introgression of a superior haplotype improving grain yield and water-nutrient uptake under DDSR using haplotype‐based breeding may open new avenues for designing next-generation DDSR-adapted rice varieties.

## Conclusions

A complex mapping population having wide phenotypic variability coupled with large genome coverage was used to identify significant marker-phenotype associations to be exploited directly in breeding programs. A total of 10 significant trait-SNP associations and 25 QTLs associated with 25 traits of interest were identified under DDSR. The colocation of the SNPs related to root morphologcal traits, nutrient uptake, and grain yield under DDSR was further confirmed by the direct significant positive correlation of root traits with nutrient uptake. The identification of suitable traits and promising progenies possessing colocated QTLs may serve as novel breeding material for developing DDSR varieties. The identification and introgression of a superior haplotype exploiting haplotype-based breeding would be the next step to improve the grain yield and adaptability of rice under DDSR.

## Materials and Methods

### Development of a complex mapping population

The plant material used in the present study comprised a complex mapping population derived from six diverse parents: IR 74371-70-1-1, UPLRi 7, IRRI 123, Kali Aus, Vandana, and IRRI 148. The detailed characterstics of the parental lines used to develop the complex mapping population are presented in Supplementary Table S2. The scheme for the development of the complex mapping population and details on the phenotyping and genotyping screening are provided in Supplementary Fig. S1.

### Details on experiments and progenies

The field experiments were conducted in the 2015 wet season (2015WS) and 2016 dry season (2016DS) in upland farm fields at IRRI (International Rice Research Institute, Los Baños, Laguna, Philippines, 14°10′11.81″N, 121°15′22″E). Seeding was done at ~2 cm depth in 3.0 m rows with two rows per progeny in dry plots. Out of the total 3 m row, the central 2 m [20 cm (hill to hill) × 20 cm (row to row)] was used for the measurment of grain yield and yield-related traits and the remaining 1.0 m [10 cm (hill to hill) × 20 cm (row to row)] was used for the destructive sampling for the root traits at different growth points (15, 22, and 29 DAS). A total of 450 F_2_-derived F_3_ progenies and 300 F_3_-derived F_4_ progenies along with six parents and four checks were evaluated in 2015WS and 2016DS, respectively, under DDSR conditions. In 2015WS, the field evaluations were carried out in an augmented design with 450 F_3_ progenies, six parents, and four checks (100 progenies per block and a total of 5 blocks); whereas, in 2016DS, the evaluations involved 300 lines, six parents, and four checks in an alpha-lattice design (5 progenies per block and a total of 62 blocks and 2 replications).

### Soil characteristics

The sand, silt, and clay content of the experimental soil was 21%, 36%, and 39%, respectively, with pH 7.7. The Ca (calcium), Mg (magnesium), K (potassium), P (phosphorus), and KjN (Kjeldahl nitrogen) content was 16.0 meq (milliequivalents) 100 g^−1^, 7.8 meq 100 g^−1^, 1.09 meq 100 g^−1^, 20 mg kg^−1^, and 0.10%, respectively. The data on average rainfall, temperature, humidity, solar radiation, and pressure across 2015WS and 2016DS are presented in Supplementary Fig. S2.

### Details on field managment

The term “dry direct-seeded” used in the present study refers to the cultivation method in which the rice crop is seeded directly in the dry field as wheat and corn (maize) without any nursery-bed raising in non-puddled, laser-leveled fields.

To ensure better pulverization, uniform germination, and, most importantly, weed control under DDSR, the field was prepared 1 month prior to sowing. Land preparation involved plowing using a terra disc plow followed by three rotovations at weekly intervals. The field was laser-leveled and allowed for the first flush of weeds to emerge and grow for 3 weeks, before control with the application of glyphosate (1.0 kg ai ha^−1^; ai: active ingredients). A combination of pre-emergence (oxadiazon at 0.5 kg ai ha^−1^ at 6 DAS), early post-emergence (bispyribac sodium at 0.03 kg ai ha^−1^ (9.7%, Nominee) at 11 and 22 DAS), and spot weeding at 35 and 55 DAS was used to control weeds.

During the seedling-establishment stage, sprinkler irrigation was used for 1 month and thereafter surface irrigation was applied once or twice a week depending on the weather and crop water status. The irrigated field was allowed to drain naturally through normal seepage and percolation.

The total fertilizer rate was 100-35-30 N-P-K kg ha^−1^ and 120-40-40 N-P-K kg ha^−1^ in 2015WS and 2016DS, respectively. A detailed description of the experiments conducted and traits measured in the present study is presented in Supplementary Table S3.

### Phenotypic evaluation of the complex mapping population

#### Measurement of seedling establishment traits

Each plot was monitored regularly for first and full emergence from 3 to 12 DAS. The term “days to first emergence” used in the present study refers to the emergence of one or two seedlings from the soil and “days to full emergence” refers to the emergence of 90–95% of the seedlings per plot.

Vegetative vigor in terms of relative growth rate (RGR) was recorded from three randomly selected seedlings per plot. The seedlings were uprooted using a trowel at 15, 22, and 29 DAS. The roots and shoots were separated and the shoots were oven-dried at 60 °C for 72 hours with each sampling. The oven-dried shoots were weighed (DSW) immediately after being taken out from the oven to calculate RGR. RGR was calculated at different timepoints (i.e., from 15 to 22 DAS (RGR1), 22 to 29 DAS (RGR2), and 15 to 29 DAS (RGR3)) using the following equation^4^:

$$\frac{\mathrm{log}\,({\rm{dry}}\,{\rm{shoot}}\,{\rm{weight}}\,{\rm{at}}\,{\rm{sampling}}\,2)-\,\mathrm{log}\,({\rm{dry}}\,{\rm{shoot}}\,{\rm{weight}}\,{\rm{at}}\,{\rm{sampling}}\,{\rm{1}})}{({\rm{date}}\,{\rm{of}}\,{\rm{sampling}}\,2-{\rm{date}}\,{\rm{of}}\,{\rm{sampling}}\,{\rm{1}})}$$

The separated root samples were used for the root trait measurements. At 30 DAS, VVG was also recorded on a 1‒9 scale (1: extra vigorous, 3: vigorous, 5: normal, 7: weak, 9: very weak) following the IRRI Standard Evaluation System for Rice^65^.

#### Measurement of root traits

Randomly, three plants were removed from the soil at each sampling to measure root traits. The soil sections surrounding the plant containing roots were removed by digging a hole (40 cm deep). The roots and shoots were then separated by cutting from the topsoil line. The separated root samples at 15, 22, and 29 DAS were washed gently and properly with running tap water on a sieve and root fresh weight (RFW in g) was measured quickly. The number of nodal roots (NR) [NR1 (number of nodal roots at 15 DAS), NR2 (number of nodal roots at 22 DAS), NR3 (number of nodal roots at 29 DAS)] was counted manually. Maximum root length (RL) [RL1 (maximum root length at 15 DAS), RL2 (maximum root length at 22 DAS), RL3 (maximum root length at 29 DAS)] was measured using the centimeter scale. The roots were then oven-dried at 60 °C for 72 hours for root dry weight (RDW) measurements. Root hair length (RHL) and root hair density (RHD) were recorded on six root samples following the procedure as described by Sandhu *et al*.^4^.

At booting stage, the color of the central part of the middle lobe of three randomly chosen flag leaves was compared with the color strip (LCC: leaf color chart developed by IRRI); to validate the greenness of fully expanded flag leaves, a Minolta 502 chlorophyll meter (SPAD) was used. To estimate the correlation with nitrogen concentration and N uptake, total aboveground fresh biomass was measured immediately after uprooting the same three plants. Then, the plant samples from each progeny were cut into small pieces, mixed properly, and a total of 200 g of plant sample were taken, oven-dried at 70 °C for 3 days, and then weighed immediately for dry biomass. A total of 60 progenies (30 high-yielding and 30 low-yielding and parents) were selected in 2015WS and analyzed for nutrient uptake (N, P, Fe, and Zn) in the IRRI Analytical Service Laboratory. The nitrogen estimation was carried out using the Kjeldahl digestion method. The plant material was digested with a mixture of K_2_SO_4_ (potassium sulfate) and concentrated H_2_SO_4_ (sulfuric acid) in the presence of a catalyst (fine-powdered selenium). The NH_3_ (ammonia) produced was estimated by the colorimetric method using a Technicon autoanalyzer. The other nutrients phosphorus (P), iron (Fe), and zinc (Zn) were estimated using the nitric/perchloric acid digestion method using the AIM 500 Digestion Block System.

#### Lodging resistance traits

The lodging resistance traits stem diameter (SD; mm), culm diamter (CD; mm), bending strength (BS; kg cm), and bending moment (BM, kg cm^2^) were measured on three randomly chosen plants. SD and CD were measured using a Vernier caliper. A prostrate tester (Daiki Rika Kogyou Co., Tokyo)^66^ was used to measure stem strength. Twenty days after flowering, the main stem of each randomly chosen plant was cut from the ground level and force was given to the second and third internode of the stem at the middle (5 cm) of the internode and the bending ability of the internode was measured by bending the stem to the point at which the stem broke. The displacement in mm was measured as stem strength at the breaking point. The scale displacement (mm) on the prostrate tester due to bending ability was recorded.

#### Plant morphological, grain yield, and yield-related traits

Five plants were randomly chosen to record data on plant morphological, grain yield, and yield-related traits. The plant morphological traits cover flag-leaf length (FLL), flag-leaf width (FLW), flag-leaf area (FLA), flag-leaf angle (FLAngle), and plant height (PHT). The grain yield and yield-related traits include days to 50% flowering (DTF), number of productive tillers per plant (NPT), panicle length (PL), filled grains per panicle (FG/P), 1000-grain weight (1000 GW), biomass at flowering (BMF), and straw yield (SY). DTF was recorded when ~50% of the plants in a plot showed panicle exsertion. PHT, PL, FLL, and FLW were measured on a centimeter scale (cm). PHT was measured from the ground level to the tip of the highest panicle at maturity stage. NPT was counted manually. Panicle length was measured from the node of the panicle to the tip of the panicle. FLL was measured from the base to the tip of the flag leaf whereas FLW was measured at the middle part of the flag leaf. Flag-leaf area (FLA; cm^2^) was calculated according to Palamiswamy and Gomez^67^. FLAngle was measured using a protector keeping the stem as the horizontal base. The plants were harvested at hard dough stage and the grains and straw were separated. The harvested grains were threshed, cleaned, and oven-dried for 3 days at 50 °C to 14% mositure content and then weighed to record grain yield. The filled grains per panicle were counted and 1000-grain weight was recorded in g. For straw yield, the harvested straw was oven-dried for 3 days at 70 °C and weighed. Biomass at flowering (BMF) was measured (in g) after harvesting and drying the aboveground biomass at 70 °C in an oven till there was no change in the dry weight of the plants.

#### DNA isolation, genotyping by sequencing, and SNP calling

The samples for genotyping comprised six parents, four checks, and 300 progenies from the complex mapping population in 2016DS. The fresh leaf tissue samples from six plants per progeny were collected at 40 DAS. Automated leaf sampling with high-throughput DNA extraction using the Brooks’ PlantTrak Hx rice leaf tissue sampler and LGC Genomics’ oKtopure systems, respectively, was performed to increase throughput and efficiency coupled with precision. *ApeKI*, a type II restriction endonuclease, was used for DNA digestion. The digested DNAs were ligated to the adapter and 96-plex libraries were constructed as per the genotyping by sequencing (GBS) protocol. GBS was carried out using the HiSeq. 2000 100PE platform of Macrogen Inc. (Korea). From GBS data, a total of 971,790 SNPs were called from the GBS genotyping. A stringent selection criterion to filter out the SNPs was used, including missing percentage, MAF (minor allele frequency), and percent heterozygosity, to select the panel of robust SNPs. SNPs with a call rate of 85% and MAF of >5% (10,588 SNPs) were filtered using Tassel 5^68^. The 10,588 SNPs were used to estimate the genetic relationship, building of a neighbor joining (NJ) tree, and to carry out GWAS. In order to detect and correct for population structure, a PCA was carried out using 10,588 SNP markers.

#### Phenotypic data analysis

Analysis of variance (ANOVA) was computed using PBTools V 1.4.0 (http://bbi.irri.org/products). The trial mean and trait mean for each season were computed using mixed model analysis considering replications, and block within replication as a random effect and progenies as a fixed effect. Broad-sense heritability was computed using the equation

$${\rm{H}}={{\sigma }^{2}}_{{\rm{G}}}/({{\sigma }^{2}}_{{\rm{G}}}+{{\sigma }^{2}}_{{\rm{E}}}/r)$$

where H represents the broad-sense heritability, σ^2^G represents the genetic variance, σ^2^E the error variance, and r the number of replications. Correlation among traits was calculated using function rcorr() [in Hmisc package] in R. v.1.1.423.

The best parent for the promising traits was selected and the percent improvement in selected promising breeding progenies over the best parent for each promising trait was calculated as % improvement = (trait value in selected promising progeny − trait value in best parent/trait value in best parent) *100.

#### Population structure, linkage disequilibrium, and association analysis

A total of 10,558 SNPs, 300 progenies, and 6 parents were used to access the population structure using STRUCTURE V. 2.3.4 software. A series of models with *K* value ranging from 1 to 10 was run with a burn-in period to 50,000 and running length to 10,000 to give consistent results over runs. A total of 10 independent runs for each *K* were performed to verify the consistency and accuracy of the results. The most probable number of clusters was determined by the *K* value with maximum likelihood over the runs^69^. The number of principal components (PC) explaining genetic variation was estimated using R/GAPIT and iteratively added to the fixed part of the model, ranging from PC1 to PC10.

The distance matrix was estimated using Tassel 5.0^68^ and FigTree v1.4.2^70^ was used to visualize an unweighted neighbor joining tree. Tassel 5.0 was used to calculate the pairwise r^2^ values. These pairwise r^2^ values were averaged over the SNPs grouped based on stepwise increasing base-pair distance (0–10, 10–50, 50–500, 500–1000, 1000–1500, 1500–2000, 2000–2500, 2500–3000, 3000–3500, 3500–4000, 4000–4500, and 4500–5000). The LD (linkage disequilibrium) decay in the complex mapping population was estimated from the average LD over the calculated grouped base-pair distance in the complex mapping population. The genome-wide significant associations of genomic regions and traits of interest were identified using CMLM (compressed mixed linear model)/P3D (population parameters previously defined) in GAPIT (Genome Association and Prediction Integrated Tool) executed by R package^71^ on 2015WS, 2016DS, and the combined data over seasons.

Genetic similarity between individuals was estimated using the relatedness matrix and the random effects were estimated from identical by state (IBS) values. The population structure (*Q* value) and kinship matrix (*K*) estimated from the genotyping data were used to improve the statistical power of genome-wide association mapping. The allelic effect of the significant markers associated with the trait of interest in the present study was determined by representing the phenotypic data for the alleles as boxplots and the significant allelic variation for the associated trait of interest was determined performing the Kruskal–Wallis test in “R”.

#### Correction of false discovery rate (FDR)/Bonferroni corrections

To correct false positives in genome-wide association analysis even keeping a stringent p-value benchmark, the Bonferroni correction method was used. After the Bonferroni multiple test correction (0.01/10588; significance level of 1%/total number of markers used in analysis), the calculated threshold value was 9.4 × 10^−7^.

#### Prediction of putative candidate genes in the identified genomic region

On average across diverse rice germplasm, LD decays half of its maximum value in the 100-kb region. The putative candidate genes in the 100-kb region (100 kb upstream and 100 kb downstream region of the SNPs with significant association signals) were searched using MSU v.7 rice genome browser (http://rice.plantbiology.msu.edu/cgi-bin/gbrowse/rice/#search), QTARO database (http://qtaro.abr.affrc.go.jp), and the existing literature.

## Supplementary information

Supplementary dataset

## References

[1] Thanawong K, Perret SR, Basset-Mens C (2014). Eco-efficiency of paddy rice production in Northeastern Thailand: a comparison of rain-fed and irrigated cropping systems. J. Cle. Prod..

[2] Bouman, B. A. M., Wang, H., Yang, X., Zhao, J. & Wang, C. Aerobic rice (Han Dao): a new way of growing rice in water-short areas. In *Proceedings of the 12th international soil conservation organization conference* (Vol. 26, p. 31). Beijing, China: Tsinghua University Press (2002, May).

[3] Kumar V, Ladha JK (2011). Direct seeding of rice: recent developments and future research needs. In Adv. Agron..

[4] Sandhu N (2015). Traits and QTLs for development of dry direct-seeded rainfed rice varieties. J. Exp. Bot..

[5] Yano K (2015). Isolation of a novel lodging resistance QTL gene involved in strigolactone signaling and its pyramiding with a QTL gene involved in another mechanism. Mol. Plant.

[6] Comas L, Becker S, Cruz VMV, Byrne PF, Dierig DA (2013). Root traits contributing to plant productivity under drought. Front. Plant Sci..

[7] Sandhu N (2016). Rice root architectural plasticity traits and genetic regions for adaptability to variable cultivation and stress conditions. Plant Physiol..

[8] Shahzad Z, Amtmann A (2017). Food for thought: how nutrients regulate root system architecture. Curr. Opin. Plant Biol..

[9] Zhu XG, Long SP, Ort DR (2010). Improving photosynthetic efficiency for greater yield. Ann. Rev. Plant Biol..

[10] Horton P (2000). Prospects for crop improvement through the genetic manipulation of photosynthesis: morphological and biochemical aspects of light capture. J. Exp. Bot..

[11] Yano M, Kojima S, Takahashi Y, Lin H, Sasaki T (2001). Genetic control of flowering time in rice, a short-day plant. Plant Physiol..

[12] Xue W (2008). Natural variation in Ghd7 is an important regulator of heading date and yield potential in rice. Nat. Genet..

[13] Yan WH (2011). A major QTL, *Ghd8*, plays pleiotropic roles in regulating grain productivity, plant height, and heading date in rice. Mol. Plant.

[14] Spielmeyer W, Ellis MH, Chandler PM (2002). Semidwarf (sd-1),“green revolution” rice, contains a defective gibberellin 20-oxidase gene. Proc. Nat. Acad. Sci..

[15] Han J (2015). ZD958 is a low-nitrogen-efficient maize hybrid at the seedling stage among five maize and two teosinte lines. Planta.

[16] Varshney RK, Graner A, Sorrells ME (2005). Genomics-assisted breeding for crop improvement. Trends Plant Sci..

[17] Varshney RK, Terauchi R, McCouch SR (2014). Harvesting the promising fruits of genomics: applying genome sequencing technologies to crop breeding. PLoS Biol..

[18] Kang YJ (2016). Translational genomics for plant breeding with the genome sequence explosion. Plant Biotechnol. J..

[19] Yu J, Holland JB, McMullen MD, Buckler ES (2008). Genetic design and statistical power of nested association mapping in maize. Genetics.

[20] Zhu C, Gore M, Buckler ES, Yu J (2008). Status and prospects of association mapping in plants. Plant Gene.

[21] Flint-Garcia SA, Thornsberry JM, Buckler ES (2003). Structure of linkage disequilibrium in plants. Ann. Rev. Plant Biol..

[22] Schmid M, Bennewitz J (2017). Invited review: Genome-wide association analysis for quantitative traits in livestock–a selective review of statistical models and experimental designs. Arc. Anim. Breed..

[23] Rafalski JA (2010). Association genetics in crop improvement. Curr. Opin. Plant Biol..

[24] Zhao K (2011). Genome-wide association mapping reveals a rich genetic architecture of complex traits in *Oryza sativa*. Nat. Comm..

[25] Huang X, Han B (2014). Natural variations and genome-wide association studies in crop plants. Ann. Rev. Plant Biol..

[26] Yang W (2014). Combining high-throughput phenotyping and genome-wide association studies to reveal natural genetic variation in rice. Nat. Comm..

[27] Zhang Z (2014). Improving the accuracy of whole genome prediction for complex traits using the results of genome wide association studies. PloS One.

[28] Korte A, Farlow A (2013). The advantages and limitations of trait analysis with GWAS: a review. Plant Met..

[29] Wilson LM (2004). Dissection of maize kernel composition and starch production by candidate gene association. Plant Cell.

[30] Camus-Kulandaivelu L (2006). Maize adaptation to temperate climate: relationship with population structure and polymorphism in the Dwarf8 gene. Genetics.

[31] Jun TH, Van K, Kim MY, Lee SH, Walker DR (2008). Association analysis using SSR markers to find QTL for seed protein content in soybean. Euphytica.

[32] Jia G (2013). A haplotype map of genomic variations and genome-wide association studies of agronomic traits in foxtail millet (*Setaria italica*). Nat. Genet..

[33] Kale SM (2015). Prioritization of candidate genes in “QTL-hotspot” region for drought tolerance in chickpea (*Cicer arietinum* L.). Sci. Rep..

[34] Saxena RK (2017). Construction of genotyping-by-sequencing based high-density genetic maps and QTL mapping for fusarium wilt resistance in pigeonpea. Sci. Rep..

[35] Saxena RK (2017). Genotyping-by-sequencing of three mapping populations for identification of candidate genomic regions for resistance to sterility mosaic disease in pigeonpea. Sci. Rep..

[36] Joshi E (2013). Management of direct seeded rice for enhanced resource-use efficiency. Plant Know. J..

[37] Yu LX (2011). Association mapping and gene–gene interaction for stem rust resistance in CIMMYT spring wheat germplasm. Theor. Appl. Genet..

[38] Huang X (2010). Genome-wide association studies of 14 agronomic traits in rice landraces. Nat. Genet..

[39] Komatsu K (2003). LAX and SPA: major regulators of shoot branching in rice. Proc. Nat. Acad. Sci..

[40] Koumoto T (2013). Rice monoculm mutation moc2, which inhibits outgrowth of the second tillers, is ascribed to lack of a fructose-1, 6-bisphosphatase. Plant Biotechnol..

[41] Cho SH, Yoo SC, Zhang H, Lim JH, Paek NC (2014). Rice NARROW LEAF1 regulates leaf and adventitious root development. Plant Mol. Biol. Rep..

[42] Kamoshita A (2002). Mapping QTLs for root morphology of a rice population adapted to rainfed lowland conditions. Theor. Appl. Genet..

[43] Courtois B (2003). Locating QTLs controlling constitutive root traits in the rice population IAC 165 × Co39. Euphytica.

[44] Qu Y (2008). Mapping QTLs of root morphological traits at different growth stages in rice. Genetica.

[45] Sakamoto T (2006). Ectopic expression of KNOTTED1-like homeobox protein induces expression of cytokinin biosynthesis genes in rice. Plant Physiol..

[46] Koiwai H (2007). RING‐H2 type ubiquitin ligase EL5 is involved in root development through the maintenance of cell viability in rice. Plant J..

[47] Liu W, Xu ZH, Luo D, Xue HW (2003). Roles of OsCKI1, a rice casein kinase I, in root development and plant hormone sensitivity. Plant J..

[48] Yan M (2011). Rice OsNAR2.1 interacts with OsNRT2.1, OsNRT2.2 and OsNRT2.3a nitrate transporters to provide uptake over high and low concentration ranges. Plant Cell Environ..

[49] Wang Z (2014). Rice SPX1 and SPX2 inhibit phosphate starvation responses through interacting with PHR2 in a phosphate-dependent manner. Proc. Nat. Acad. Sci..

[50] Hirano K (2010). Rice BRITTLE CULM 3 (BC3) encodes a classical dynamin OsDRP2B essential for proper secondary cell wall synthesis. Planta.

[51] Monna L (2002). Positional cloning of rice semidwarfing gene, sd-1: rice “green revolution gene” encodes a mutant enzyme involved in gibberellin synthesis. DNA Res..

[52] Vikram P (2015). Drought susceptibility of modern rice varieties: an effect of linkage of drought tolerance with undesirable traits. Sci. Rep..

[53] Wang ZF, Wang JF, Bao YM, Wang FH, Zhang HS (2010). Quantitative trait loci analysis for rice seed vigor during the germination stage. J. Zhe. Univ. Sci. B.

[54] Moncada P (2001). Quantitative trait loci for yield and yield components in an *Oryza sativa* × *Oryza rufipogon* BC_2_F_2_ population evaluated in an upland environment. Theor. Appl. Genet..

[55] Wang X (2014). Phosphate transporters OsPHT1;9 and OsPHT1;10 are involved in phosphate uptake in rice. Plant Cell Environ..

[56] Nakamura H (2009). Ectopic overexpression of the transcription factor OsGLK1 induces chloroplast development in non-green rice cells. Plant Cell Physiol..

[57] Morita R, Sato Y, Masuda Y, Nishimura M, Kusaba M (2009). Defect in non‐yellow coloring 3, an α/β hydrolase‐fold family protein, causes a stay‐green phenotype during leaf senescence in rice. Plant J..

[58] Yang Y (2014). Morphological characteristics and gene mapping of a dense panicle (dp2) mutant in rice (*Oryza sativa* L.). Genes & Genomes.

[59] Nakagawa M, Shimamoto K, Kyozuka J (2002). Overexpression of RCN1 and RCN2, rice TERMINAL FLOWER 1/CENTRORADIALIS homologs, confers delay of phase transition and altered panicle morphology in rice. Plant J..

[60] Caverzan A (2014). The knockdown of chloroplastic ascorbate peroxidases reveals its regulatory role in the photosynthesis and protection under photo-oxidative stress in rice. Plant Sci..

[61] Chen L (2013). OsGRAS19 may be a novel component involved in the brassinosteroid signaling pathway in rice. Mol. Plant.

[62] Bao, Z. *et al*. A rice gene for microbial symbiosis, OsCCaMK, reduces CH4 flux in a paddy field with low nitrogen input. *Appl. Environ. Microbiol*. AEM-03646 (2014). 10.1128/AEM.03646-13 PMC3957643 24441161

[63] Miyoshi K, Ito Y, Serizawa A, Kurata N (2003). OsHAP3 genes regulate chloroplast biogenesis in rice. Plant J..

[64] Nath K (2013). Loss‐of‐function of Os STN 8 suppresses the photosystem II core protein phosphorylation and interferes with the photosystem II repair mechanism in rice (*Oryza sativa*). Plant J..

[65] IRRI. *SES* (Standard Evaluation System for Rice). International Network for Genetic Evaluation of Rice. Los Baños, Philippines: International Rice Research Institute (IRRI) (1996).

[66] Kashiwagi T, Ishimaru K (2004). Identification and functional analysis of a locus for improvement of lodging resistance in rice. Plant Physiol..

[67] Palamiswamy KM, Gomez KA (1974). Length–width method for estimating leaf area of rice. Agron. J..

[68] Bradbury PJ (2007). TASSEL: software for association mapping of complex traits in diverse samples. Bioinformatics.

[69] Pritchard, J. & Wen, W. Department of Human Genetics, University of Chicago, 920 E 58th St., CLCS 507, Chicago, IL 60637, USA (2004).

[70] Kimura M (1980). A simple method for estimating evolutionary rates of base substitutions through comparative studies of nucleotide sequences. J. Mol. Evol..

[71] Lipka AE (2012). GAPIT: genome association and prediction integrated tool. Bioinformatics.

[72] Sentoku N, Sato Y, Matsuoka M (2000). Overexpression of rice OSH genes induces ectopic shoots on leaf sheaths of transgenic rice plants. Dev. Biol..

[73] Jeong JS (2010). Root-specific expression of OsNAC10 improves drought tolerance and grain yield in rice under field drought conditions. Plant Physiol..

[74] Sakamoto T (2011). Rice CYP734As function as multisubstrate and multifunctional enzymes in brassinosteroid catabolism. Plant J..

[75] Sato K (2010). Isolation of a novel cell wall architecture mutant of rice with defective *Arabidopsis* COBL4 ortholog BC1 required for regulated deposition of secondary cell wall components. Planta.

[76] Xiao J, Li J, Yuan L, Tanksley SD (1996). Identification of QTLs affecting traits of agronomic importance in a recombinant inbred population derived from a subspecific rice cross. Theor. Appl. Genet..

[77] Cui KH, Peng SB, Xing YZ, Yu SB, Xu CG (2002). Genetic analysis of the panicle traits related to yield sink size of rice. Yi chuan xue bao = Acta Genet. Sin..

[78] Kobayashi K (2012). Inflorescence meristem identity in rice is specified by overlapping functions of three AP1/FUL-like MADS box genes and PAP2, a SEPALLATA MADS box gene. Plant Cell.

[79] Kobayashi K, Maekawa M, Miyao A, Hirochika H, Kyozuka J (2009). PANICLE *PHYTOMER2* (*PAP2*), encoding a SEPALLATA subfamily MADS-box protein, positively controls spikelet meristem identity in rice. Plant Cell Physiol..

[80] Gao X (2010). The SEPALLATA-like gene OsMADS34 is required for rice inflorescence and spikelet development. Plant Physiol..

[81] Zou Y (2014). OsRPK1, a novel leucine-rich repeat receptor-like kinase, negatively regulates polar auxin transport and root development in rice. Biol. et Biol. Acta (BBA)-Gen. Sub..

