# Supplementary material for: Deciphering the genetic basis of root morphology, nutrient uptake, yield, and yield-related traits in rice under dry direct-seeded cultivation systems
Source: Sci Rep. 2019 Jun 27;9:9334. doi: 10.1038/s41598-019-45770-3 (PMC6597570; doi:10.1038/s41598-019-45770-3)
Supplement: Supplementary file 1 — Supplementary dataset [file 41598_2019_45770_MOESM1_ESM.docx]

**Deciphering the genetic basis of root morphology, nutrient uptake, yield, and yield-related traits in rice under dry direct-seeded cultivation systems**

Nitika Sandhu^1,8^, Sushil Raj Subedi^1,6,7^, Vikas Kumar Singh^2^, Pallavi Sinha^3^, Santosh Kumar^4^, S. P. Singh^5^_,_ Surya Kant Ghimire^6^, Madav Pandey^6^, Ram Baran Yadaw^7^, Rajeev K. Varshney^3^, Arvind Kumar^1*^

1. Rice Breeding Platform, International Rice Research Institute, Metro Manila, Philippines
2. International Rice Research Institute, South Asia Hub, ICRISAT, Patancheru, Hyderabad, India
3. Center of Excellence in Genomics and System Biology, International Crops Research Institute for the Semi-Arid Tropics (ICRISAT), Patancheru, Hyderabad, India
4. ICAR Research Complex for Eastern Region, Patna, Bihar
5. Bihar Agricultural University, Sabour, Bihar
6. Agriculture and Forestry University**, Rampur, Chitwan, Nepal**
7. National Rice Research Program, Hardinath, Nepal
8. Punjab Agricultural University, Ludhiana, India

*Corresponding author: a.kumar@irri.org

^1^International Rice Research Institute, DAPO Box 7777, Metro Manila, Philippines

**Nitika Sandhu** : [n.sandhu@irri.org](mailto:n.sandhu@irri.org)

**Sushil Raj Subedi** : subedirajsubedi@gmail.com

**Vikas Kumar Singh** : v.k.singh@irri.org

**Pallavi Sinha** : p.sinha@cgiar.org

**Santosh Kumar** : [santosh9239@gmail.com](mailto:santosh9239@gmail.com)

**S. P. Singh** : [sps2007bau2011@gmail.com](mailto:sps2007bau2011@gmail.com)

**Surya Kant Ghimire** : suryaghimire2003@yahoo.com

**Madav Pandey** : mppandey@afu.edu.np

**Ram Baran Yadaw**  : rbaran_9@yahoo.com

**Rajeev K. Varshney** : [r.k.varshney@cgiar.org](mailto:r.k.varshney@cgiar.org)

**Arvind Kumar** : a.kumar@irri.org

**Corresponding author*

**Arvind Kumar**

Outcome Theme Leader-Resilient Rice

Plant Breeder, Rice Breeding Platform

International Rice Research Institute, DAPO Box 7777,

Metro Manila, Philippines

**Supplementary Table S1:** Detailed description of the candidate genes identified near or in the region of significant marker-trait associations/putative QTLs identified in the present study using MSU v.7 rice genome browser (<http://rice.plantbiology.msu.edu/cgi-bin/gbrowse/rice/#search>).

| **Traits** | **SNP** | **Pos. (MSU 7.0)** | **Locus name (MSU 7.0)** | **Gene product name** | **Gene product description** |
| --- | --- | --- | --- | --- | --- |
| Number of nodal roots at 29 DAS | S4_31728342 | 31727770 - 31729348 | LOC_Os04g53260.1 |  | polyphenol oxidase protein, putative, expressed |
|  |  | 31759581 - 31750955 | LOC_Os04g53310.1 |  | soluble starch synthase 3, chloroplast precursor, putative, expressed |
|  |  | 31693570 - 31696647 | LOC_Os04g53214.2 |  | hydroxyacid oxidase 1, putative, expressed |
|  |  | 31700907 - 31703617 | LOC_Os04g53230.1 |  | aminomethyltransferase, putative, expressed |
| Root hair length, root hair density | S5_15470847 | 15464454 - 15465082 | LOC_Os05g26620.1 | PROLM14 | prolamin precursor, putative, expressed |
|  | S5_15470880 | 15488221 - 15482300 | LOC_Os05g26660.1 |  | NADH-ubiquinone oxidoreductase, mitochondrial precursor, putative, expressed |
| Flag-leaf area | S5_15133031 | 15124584 - 15125829 | LOC_Os05g26020.1 |  | cyclin, putative, expressed |
|  |  | 15136288 - 15147503 | LOC_Os05g26040.1 |  | pumilio-family RNA binding repeat-containing protein, expressed |
|  | S4_21832119 | 21816178 - 21812157 | LOC_Os04g35790.3 |  | GLTP domain-containing protein, putative, expressed |
|  |  | 21823723 - 21817394 | LOC_Os04g35800.1 |  | zinc finger C-x8-C-x5-C-x3-H type family protein, expressed |
|  |  | 21842286 - 21848136 | LOC_Os04g35840.1 |  | T-complex protein 11, putative, expressed |
|  |  | 21858308 - 21856902 | LOC_Os04g35864.1 |  | DDT domain-containing protein, putative, expressed |
| Flag-leaf angle | S9_19178976 | 19155796 - 19150469 | LOC_Os09g32090.1 |  | methyl-binding domain protein MBD, putative, expressed |
| Days to first emergence | S6_16444037, S6_16444038, S6_16444082 | 16400699 - 16432410 | LOC_Os06g28820.1 |  | cycloartenol synthase, putative, expressed |
| Days to full emergence | S11_24806565, S11_24806596, S11_24806601 | 24809640 - 24810818 | LOC_Os11g41350.1 | MBTB67 | Bric-a-Brac, Tramtrack, Broad Complex BTB domain with Meprin and TRAF Homology MATH domain, expressed |
|  |  | 24750529 - 24751632 | LOC_Os11g41290.1 | MrBTB5 | Bric-a-Brac, Tramtrack, Broad Complex BTB domain with Meprin and TRAF Homology MATH-related domain, expressed |
|  |  | 24756166 - 24757242 | LOC_Os11g41300.1 | MBTB65 | Bric-a-Brac, Tramtrack, Broad Complex BTB domain with Meprin and TRAF Homology MATH domain, expressed |
|  |  | 24758712 - 24759854 | LOC_Os11g41310.1 | MBTB66 | Bric-a-Brac, Tramtrack, Broad Complex BTB domain with Meprin and TRAF Homology MATH domain, expressed |
| SPAD (Soil Plant Analysis Development chlorophyll meter) | S2_16013833, S2_16191048 | 16082576 - 16083406 | LOC_Os02g27300.1 |  | SCP-like extracellular protein, expressed |
|  |  | 16088547 - 16094120 | LOC_Os02g27310.1 | TKL_IRAK_DUF26-lc.6 | TKL_IRAK_DUF26-lc.6 - DUF26 kinases have homology to DUF26-containing loci, expressed |
|  |  | 16120440 - 16114897 | LOC_Os02g27360.1 |  | aspartic proteinase-like protein 2 precursor, putative, expressed |
|  |  | 16150035 - 16152929 | LOC_Os02g27400.1 | OsFBX49 | F-box domain-containing protein, expressed |
|  |  | 16160890 - 16160465 | LOC_Os02g27430.1 |  | GRF zinc finger family protein |
|  |  | 16172700 - 16180337 | LOC_Os02g27440.2 |  | DNA topoisomerase 1, putative, expressed |
|  |  | 16208331 - 16216477 | LOC_Os02g27470.1 | importin-7 | putative, expressed |
| Bending strength | S3_21737519 | 21701041 - 21703525 | LOC_Os03g39040.1 |  | zinc knuckle domain-containing protein, expressed |
|  |  | 21708299 - 21709740 | LOC_Os03g39050.1 |  | no apical meristem protein, expressed |
|  |  | 21726158 - 21727660 | LOC_Os03g39100.1 |  | no apical meristem protein, expressed |
|  |  | 21732988 - 21738085 | LOC_Os03g39129.1 |  | frigida, putative, expressed |
|  |  | 21746049 - 21747104 | LOC_Os03g39150.1 |  | protein kinase domain-containing protein, expressed |
|  |  | 21748596 - 21751688 | LOC_Os03g39160.1 |  | frigida, putative, expressed |
| Plant height | S11_17805610 | 17785138 - 17792672 | LOC_Os11g30560.1 |  | dehydrogenase/reductase, putative, expressed |
|  | S1_38481437 | 38398517 - 38401533 | LOC_Os01g66120.1 |  | no apical meristem protein, putative, expressed |
|  |  | 38443627 - 38446431 | LOC_Os01g66180.1 |  | cytochrome c, putative, expressed |
| Grain yield | S11_17412133, S11_17412134, S11_17412139 | 17385302 - 17393700 | LOC_Os11g29920.1 |  | NB-ARC domain-containing protein, expressed |
|  |  | 17383893 - 17384607 | LOC_Os11g29910.1 |  | plastocyanin-like domain-containing protein, putative, expressed |
|  |  | 17403407 - 17404271 | LOC_Os11g29945.1 |  | BNR/Asp-box repeat family protein, putative, expressed |
|  |  | 17417481 - 17411995 | LOC_Os11g29970.1 |  | NB-ARC domain-containing protein, expressed |
|  |  | 17429007 - 17425545 | LOC_Os11g29980.1 |  | disease resistance RPP13-like protein 1, putative, expressed |
|  |  | 17438286 - 17434258 | LOC_Os11g29990.1 |  | NBS-LRR type disease resistance protein, putative, expressed |
| Straw yield | S11_15708717 | 15637696 - 15656956 | LOC_Os11g27170.1 | OsSCP58 | putative serine carboxypeptidase homologue, expressed |
|  |  | 15667656 - 15670726 | LOC_Os11g27240.1 |  | pentatricopeptide repeat domain-containing protein, putative, expressed |
|  |  | 15683051 - 15694859 | LOC_Os11g27264.2 | OsSCP60 | putative serine carboxypeptidase homologue, expressed |
|  |  | 15706797 - 15738377 | LOC_Os11g27329.1 | OsSCP62 |  |
|  |  | 15746593 - 15748178 | LOC_Os11g27370.1 |  | UDP-glucoronosyl and UDP-glucosyl transferase domain-containing protein, expressed |
| Days to 50% flowering | S11_17316419 | 17259927 - 17264943 | LOC_Os11g29750.1 |  | plasma membrane ATPase, putative, expressed |
|  |  | 17284028 - 17285405 | LOC_Os11g29780.1 |  | plant-specific domain TIGR01627 family protein, expressed |
|  |  | 17291100 - 17294288 | LOC_Os11g29790.1 |  | receptor kinase, putative, expressed |
|  |  | 17329608 - 17332992 | LOC_Os11g29850.1 |  | ABC transporter, ATP-binding protein, putative, expressed |
| Vegetative vigor | S1_37639734, S1_37653811 | 37616704 - 37622095 | LOC_Os01g64810.2 |  | zinc finger DHHC domain-containing protein, putative, expressed |
|  |  | 37625795 - 37636034 | LOC_Os01g64820.1 | POLA1 | putative DNA polymerase alpha catalytic subunit, expressed |
|  |  | 37637212 - 37639865 | LOC_Os01g64830.1 |  | aspartic proteinase nepenthesin precursor, putative, expressed |
|  |  | 37647019 - 37649292 | LOC_Os01g64850.1 | OsSub10 | putative subtilisin homologue, expressed |
|  |  | 37664117 - 37667349 | LOC_Os01g64890.1 |  | CorA-like magnesium transporter protein, putative, expressed |
|  |  | 37681708 - 37666708 | LOC_Os01g64900.1 | HEAT | putative, expressed |
|  |  | 37684066 - 37682288 | LOC_Os01g64910.1 |  | anthocyanidin 5,3-O-glucosyltransferase, putative, expressed |
|  |  | 37689486 - 37686056 | LOC_Os01g64920.1 |  | nuclear matrix protein 1, putative, expressed |
|  |  | 37696778 - 37699752 | LOC_Os01g64960.1 |  | chlorophyll A-B binding protein, putative, expressed |
|  | S11_25462225 | 25462998 - 25461244 | LOC_Os11g42270.1 | OsFBX432 | F-box domain-containing protein, expressed |
|  |  | 25465666 - 25464456 | LOC_Os11g42280.1 | OsFBX433 | F-box domain-containing protein, expressed |
|  |  | 25468131 - 25466557 | LOC_Os11g42290.1 |  | transferase family protein, putative, expressed |
|  |  | 25475631 - 25474150 | LOC_Os11g42300.1 | OsFBX434 | F-box domain-containing protein, expressed |
|  |  | 25478804 - 25477029 | LOC_Os11g42310.1 | OsFBL58 | F-box domain- and LRR-containing protein, expressed |
|  |  | 25492192 - 25496854 | LOC_Os11g42350.1 |  | glutathione synthetase, chloroplast precursor, putative, expressed |
|  |  | 25501586 - 25503169 | LOC_Os11g42370.1 |  | transferase family protein, putative, expressed |
| Fe uptake | S2_29221003, S2_29221015 | 29190320 - 29195573 | LOC_Os02g47744.1 |  | MYB family transcription factor, putative, expressed |
|  |  | 29199147 - 29204368 | LOC_Os02g47760.1 |  | AAA-type ATPase family protein, putative, expressed |
|  |  | 29208132 - 29206032 | LOC_Os02g47770.1 |  | ZF-HD protein dimerization region-containing protein, expressed |
|  |  | 29226327 - 29223873 | LOC_Os02g47780.1 |  | hydrolase, alpha/beta fold family domain-containing protein, expressed |
|  |  | 29229081 - 29226628 | LOC_Os02g47790.1 |  | monodehydroascorbate reductase, putative, expressed |
|  |  | 29241762 - 29238064 | LOC_Os02g47810.1 |  | dof zinc finger domain-containing protein, putative, expressed |
|  |  | 29265497 - 29260063 | LOC_Os02g47840.1 |  | universal stress protein domain-containing protein, putative, expressed |
|  |  | 29270056 - 29274644 | LOC_Os02g47850.1 |  | class I glutamine amidotransferase, putative, expressed |
|  |  | 29278137 - 29283921 | LOC_Os02g47860.2 |  | uridine kinase, putative, expressed |
|  |  | 29291634 - 29287705 | LOC_Os02g47880.1 |  | tetratricopeptide repeat domain-containing protein, expressed |
| N uptake, P uptake | S5_14987295 | 14991579 - 14993800 | LOC_Os05g25770.1 | WRKY45 | expressed |
|  |  | 14997859 - 15000770 | LOC_Os05g25780.1 |  | rhodanese-like domain-containing protein, putative, expressed |
|  |  | 15040797 - 15044759 | LOC_Os05g25840.3 |  | ELMO/CED-12 family protein, putative, expressed |
|  |  | 15046730 - 15051515 | LOC_Os05g25850.1 |  | superoxide dismutase, mitochondrial precursor, putative, expressed |
|  |  | 15056028 - 15054708 | LOC_Os05g25870.1 |  | N-dimethylguanosine tRNA methyltransferase, putative, expressed |
|  |  | 14899987 - 14904546 | LOC_Os05g25640.1 |  | cytochrome P450, putative, expressed |
| P uptake | S6_27868961, S6_30887442 | 27848256 - 27844654 | LOC_Os06g45960.1 |  | cytochrome P450, putative, expressed |
|  |  | 27831317 - 27830246 | LOC_Os06g45950.1 | OsSAUR25 | auxin-responsive SAUR gene family member, expressed |
|  |  | 27862248 - 27862649 | LOC_Os06g45970.1 | OsSAUR26 | auxin-responsive SAUR gene family member, expressed |
|  |  | 27866741 - 27874406 | LOC_Os06g45980.1 |  | toprim domain-containing protein, putative, expressed |
|  |  | 27875114 - 27877420 | LOC_Os06g45990.2 |  | patellin-5, putative, expressed |
|  |  | 27880145 - 27877522 | LOC_Os06g46000.1 |  | tubulin/FtsZ domain-containing protein, putative, expressed |
|  |  | 29072361-29076031 | LOC_Os06g0695800.1 |  | phosphate transport system permease protein 1 domain-containing protein |
|  |  | 29208089 - 29210889 | LOC_Os06g0698300.01 |  | protein phosphatase 2C family protein |
|  |  | 29383237 - 29385814 | LOC_Os06g0699200.1 |  | metallophosphoesterase domain-containing protein |
|  |  | 29508897 - 29515165 | LOC_Os06g070120.1 |  | UTP glucose-1-phosphate uridylyltransferase family protein |
|  |  | 29762530 - 29766745 | LOC_Os06g0704700.1 |  | NAD(P)-binding domain-containing protein |
|  |  | 30797115 - 30806042 | LOC_Os06g072470.1 |  | phosphatidylinositol 3- and 4-kinase catalytic domain-containing protein |
|  |  | 30866013 - 30866879 | LOC_Os06g51000.1 |  | carrier, putative, expressed |
|  |  | 30877993 - 30870317 | LOC_Os06g51029.1 | OsFtsH1 | OsFtsH1 FtsH protease, homologue of AtFtsH1/5, expressed |
|  |  | 30905815 - 30897376 | LOC_Os06g51084.1 |  | 1,4-alpha-glucan-branching enzyme, chloroplast precursor, putative, expressed |
|  |  | 30913463 - 30907000 | LOC_Os06g51100.2 |  | transmembrane protein, putative, expressed |
|  |  | 30918120 - 30915029 | LOC_Os06g51110.2 |  | cyclin, putative, expressed |
|  |  | 30936612 - 30935754 | LOC_Os06g51140.1 |  | ZOS6-11-C2H2 zinc finger protein, expressed |
|  |  | 30944196 - 30948366 | LOC_Os06g51150.1 |  | catalase isozyme B, putative, expressed |
|  |  | 30951470 - 30948619 | LOC_Os06g51160.1 |  | glycosyl transferase family 8, putative, expressed |
| Zn uptake | S7_26042045 | 26024756 - 26019539 | LOC_Os07g43470.1 |  | GTP-binding protein, putative, expressed |
|  |  | 26047492 - 26044540 | LOC_Os07g43540.1 |  | ORC6 - putative origin recognition complex subunit 6, expressed |
|  |  | 26077154 - 26075299 | LOC_Os07g43580.1 |  | MYB family transcription factor, putative, expressed |
|  |  | 25998785 - 26003517 | LOC_Os07g43420.1 |  | MYB family transcription factor, putative, expressed |
|  |  | 26017822 - 26014754 | LOC_Os07g43460.1 |  | sphingolipid C4-hydroxylase SUR2, putative, expressed |
|  |  | 26047492 - 26044540 | LOC_Os07g43540.1 | ORC6 | putative origin recognition complex subunit 6, expressed |

**Supplementary Table S2.** Characteristics of the six parental varieties used for the crossing scheme to develop the complex mapping population.

| S. no. | Parental line | Origin | Agronomic relevance |
| --- | --- | --- | --- |
| 1 | IR74371-70-1-1 | IRRI | High-yielding drought-tolerant rice variety (3.7-5.5 t ha^-1^) released as Sahod Ulan 1 in Philippines in 2009 (110 days to maturity) |
| 2 | Vandana | India | Semi-tall, white kernels with long and bold grain, upland-adaptated rice variety |
| 3 | IRRI 123 | IRRI | High-yielding irrigated variety (5-8 t ha^-1^), 105 to 110 days to maturity |
| 4 | IRRI 148 | IRRI | High-yielding irrigated variety (5-8 t ha^-1^), 105 to 110 days to maturity, improved drought-tolerant variety released in India as Sahbhagi Dhan in 2010 |
| 5 | Kali Aus | India | Medium-duration drought-tolerant variety, long and deep root system |
| 6 | UPLRi 7 | IRRI | High-yielding (4-6 t ha^-1^) upland-adapted drought-tolerant cultivar, 105 to 110 days to maturity |

**Supplementary Table S3.** Details of the experiments conducted and traits measured under dry direct-seeded conditions in 2015WS and 2016DS.

| S. no. | Year/  season | Date of seeding | No. of progenies | Parents and checks^a^ | Observed traits^b^ |
| --- | --- | --- | --- | --- | --- |
| 1 | 2015WS | June 19, 2015 | 500  (450 + 50) | IR74371-70-1-1, Vandana, IRRI 123, IRRI 148, Kali Aus, UPLRi 7, IR94225-B-82-B  IR94226-B-177-B  IR91648-B-32-B  IR91648-B-153-B | Days to first emergence, days to full emergence, number of nodal roots at 15 DAS, number of nodal roots at 22 DAS, number of nodal roots at 29 DAS, maximum root length (cm) at 15 DAS, maximum root length (cm) at 22 DAS, maximum root length (cm) at 29 DAS, dry shoot weight at 15 DAS (g), dry shoot weight at 22 DAS (g), dry shoot weight at 29 DAS (g), relative growth rate from 15 to 22 DAS, relative growth rate from 22 to 29 DAS, relative growth rate from 15 to 29 DAS, root hair length, root hair density, flag-leaf length, flag-leaf width, flag-leaf area, flag-leaf angle, leaf color chart, cholorophyll content (SPAD), stem diameter, culm diameter, bending strength (kg cm**)**, bending moment (kg cm^-2^**)**, plant height (cm), days to 50% flowering, biomass at 50% flowering (g), vegetative vigor score, number of productive tillers, panicle length (cm), number of filled grains/panicle, 1000-grain weight (g), straw yield (kg ha^-1^), grain yield (kg ha^-1^), N uptake (kg ha^-1^), P uptake (kg ha^-1^), Fe uptake (kg ha^-1^), Zn uptake (kg ha^-1^) |
| 2 | 2016DS | Dec. 23, 2015 | 310 (300 + 10) |  | Days to first emergence, days to full emergence, number of nodal roots at 15 DAS, number of nodal roots at 22 DAS, number of nodal roots at 29 DAS, maximum root length (cm) at 15 DAS, maximum root length (cm) at 22 DAS, maximum root length (cm) at 29 DAS, dry shoot weight at 15 DAS (g), dry shoot weight at 22 DAS (g), dry shoot weight at 29 DAS (g), relative growth rate from 15 to 22 DAS, relative growth rate from 22 to 29 DAS, relative growth rate from 15 to 29 DAS, root hair length, root hair density, flag-leaf length, flag-leaf width, flag-leag area, flag-leaf angle, leaf color chart, cholorophyll content (SPAD), stem diameter, culm diameter, bending strength (kg cm**)**, bending moment (kg cm^-2^), plant height (cm), days to 50% flowering, biomass at 50% flowering (g), vegetative vigor score, number of productive tillers, panicle length (cm), number of filled grains/panicle, 1000-grain weight (g), straw yield (kg ha^-1^), grain yield (kg ha^-1^) |

*WS, wet season; DS, dry season; DAS: days after seeding.*

*^a^IR94225-B-82-B (Aus276/3*IR64-derived progenies with better root traits and higher grain yield under direct-seeding conditions; Sandhu et al.^4^) IR94226-B-177-B (Aus276/3*MTU1010-derived progenies with better root traits and higher grain yield under direct-seeding conditions; Sandhu et al.^4^), IR91648-B-32-B (Moroberekan/3* Swarna-derived progenies with genetic region for early and uniform germination characteristics), IR91648-B-153-B (Moroberekan/3* Swarna-derived progenies with genetic region for lodging resistance).*


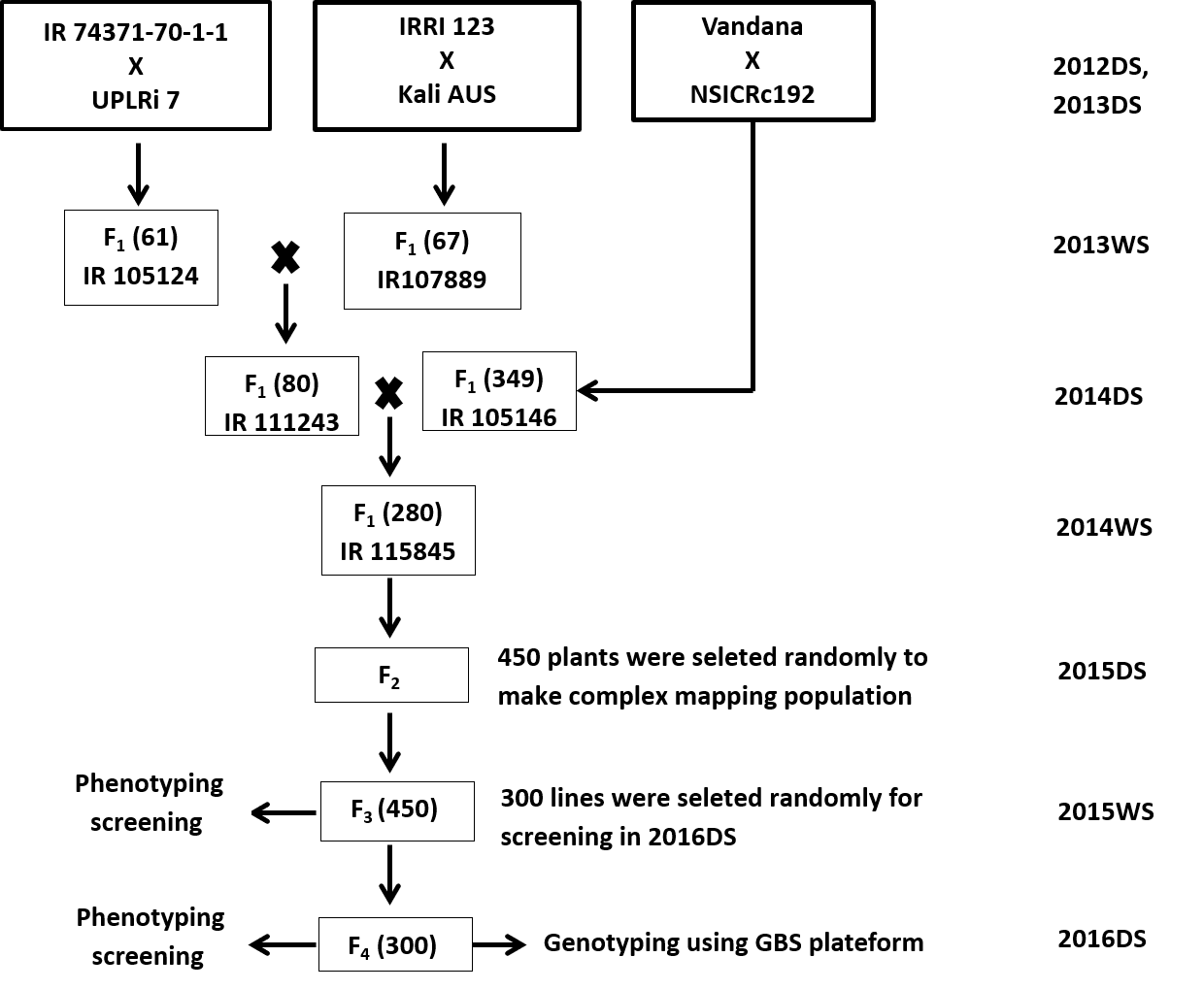


**Supplementary Figure S1.** The breeding scheme for the development of a complex mapping population involving six parents and the number of plants obtained/selected in each generation for phenotyping under direct-seeded conditions and for genotyping using the Genotyping by Sequencing platform.

**Average temperature: 28.3°C**

**Average humidity: 86%**

**Average solar radiation: 14.8 MJ m^-2^**

**Average vapour pressure: 3.1 kPa**

**(b)**

**(a)**

**Average temperature: 32.6°C**

**Average humidity: 83.5%**

**Average solar radiation: 16.2 MJ m^-2^**

**Average vapour pressure: 2.9 kPa**

**Supplementary Figure S2.** Average rainfall (mm), temperature, humidity, pressure, and air density data collected during (a) 2015WS and (b) 2016DS.
